# 14-CpG-Based Signature Improves the Prognosis Prediction of Hepatocellular Carcinoma Patients

**DOI:** 10.1155/2020/9762067

**Published:** 2020-01-04

**Authors:** Hong-ye Jiang, Gang Ning, Yen-sheng Wang, Wei-biao Lv

**Affiliations:** ^1^Department of Clinical Laboratory, Shunde Hospital, Southern Medical University (the First People's Hospital of Shunde), Foshan 528300, Guangdong Province, China; ^2^Department of Infectious Diseases, The Third Affiliated Hospital of Sun Yat-Sen University, Guangzhou 510630, Guangdong Province, China; ^3^Department of Environmental Health Science, Yale School of Public Health, New Haven, Connecticut, USA

## Abstract

**Background:**

Epigenetic dysregulation via alteration of DNA methylation often occurs during the development and progression of cancer, including hepatocellular carcinoma (HCC). In the past, many patterns of single-gene DNA methylation have been extensively explored in the context of HCC prognosis prediction. However, the combined model of a mixture of CpGs has rarely been evaluated. In the present study, we aimed to develop and validate a CpG-based signature model for HCC patient prognosis.

**Methods:**

Data from methylation profiling of GSE73003, GSE37988, and GSE57958 from the Gene Expression Omnibus (GEO) database and 371 HCC patients from the Cancer Genome Atlas (TCGA) were downloaded. The 371 HCC patients were randomly divided into a development cohort (*N* = 263) and a validation cohort (*N* = 108). Two algorithms, least absolute shrinkage and selection operator (LASSO) and robust likelihood-based survival analysis, were used to select the most significant CpGs associated with overall survival (OS) time and were used to develop and validate a methylation-based signature (MSH) for HCC patient prognosis. In addition, the prognostic efficacy of the MSH was compared with that of AJCC TNM classification and other CpG-based MSHs from TCGA. Finally, a nomogram incorporating the MSH and clinicopathologic factors was also developed.

**Results:**

Fourteen differential CpGs associated with OS were identified in HCC patients. The MSH, based on these 14 differential CpGs, could effectively divide HCC patients into two distinct subgroups with high risk or low risk of death (*P* < 0.0001) in the development cohort (26.35 vs 83.18 months, HR = 3.83, 95% CI: 2.56–5.90, *P* < 0.0001) and in the validation cohort (40.37 vs 107.03 months, HR = 2.23, 95% CI: 1.22–4.17, *P*=0.01). Univariate analysis showed that the MSH was significantly associated with OS, and the multivariate analysis also showed that the MSH was an independent prognostic factor for the OS of HCC patients in the two cohorts. In addition, stratified survival analysis indicated that the MSH still exhibited good prognostic value in different subgroups classified by AFP, cirrhosis, Child-Pugh A, tumor histologic grade, and AJCC stage. Moreover, time-dependent ROC analysis showed better performance of the MSH in predicting 3-year and 5-year survival of HCC patients than of AJCC stage and other CpG-based signatures from TCGA. The MSH-based nomogram also performed well in predicting 1-year, 3-year, and 5-year OS (C-index: 0.709).

**Conclusion:**

The 14-CpG-based signature is significantly associated with OS and may be used as a novel prognostic biomarker for HCC patients.

## 1. Introduction

Hepatocellular carcinoma (HCC) is predicted to have become the sixth most common cancer and the fourth leading cause of cancer-related death worldwide in 2018. Each year, an estimated 841,000 patients develop HCC, and 782,000 patients die from this disease [1]. Nevertheless, the threat of HCC has not been mitigated, as evidenced by the rapidly increasing incidence of HCC and the high recurrence rate of 50% among early-stage HCC patients after surgery [2, 3]. Late diagnosis and limited treatment options were suggested to account for the high mortality rate in advanced HCC patients [4]. Apart from working to find new treatment methods for this deadly disease, scientists are exploring new models for the early diagnosis and prevention of HCC to improve the prognosis of HCC.

It is well known that cancer genetics, including mutations and single-nucleotide polymorphisms (SNPs), and aberration of epigenetic regulation play important roles in the development and progression of HCC [5–7]. As one of the major epigenetic regulations, DNA methylation is reported to take part in the formation of many malignant tumors, including HCC [8, 9]. Mechanistically, aberrant DNA hypermethylation on the promoter region of CpG islands would result in the silencing of tumor suppressor genes, thus leading to the overexpression of oncogenes [10]. DNA hypermethylation on promoter CpG islands has been observed to be associated with the clinicopathological characteristics and prognosis of HCC patients in previous studies [11–13]. Identifying specific abnormal methylated CpGs may be of promising value for the diagnosis, prognosis, and even treatment of HCC.

The prognostic value of many single-gene DNA methylation patterns for HCC has been extensively explored. However, a combined model that includes assorted CpGs has rarely been evaluated. In the present study, we identified 14 differentially methylated CpGs related to HCC prognosis. We utilized the methylation profiling data of HCC from the Cancer Genome Atlas (TCGA) and developed a methylation-based signature for HCC (MSH) in the development cohort. Next, we validated the model in the validation cohort. Last, we compared the prognostic efficacy of MSH with that of the AJCC classification and other CpG-based MSHs from TCGA [14].

## 2. Materials and Methods

### 2.1. Ethics Statement

All data in the study were obtained from online databases, including Gene Expression Omnibus (GEO) and TCGA. Informed consent was obtained from the patients before the study. The study was also approved by the Ethics Committee of the Shunde Hospital of Southern Medical University.

### 2.2. Methylation Data Collection and Processing from GEO and TCGA

In our present study, DNA methylation profiles between primary HCC tumors and their nontumor counterparts from GSE73003 (including 20 paired tumor and nontumor tissues from Japan), GSE37988 (62 paired tumor and nontumor tissues from Taiwan), and GSE57958 (99 paired tumor and nontumor tissues from Singapore) were first obtained from GEO (https://www.ncbi.nlm.nih.gov/geo/). All three of these datasets were assessed on GPL8490 (Illumina Human Methylation 27 BeadChip). Next, GEO2R, an online software package, was used to identify differential CpGs. The cut-off criterion of differential CpGs was *P* < 0.05. To find the most significant differentially methylated markers, the top 1,000 CpGs with the lowest *P* values of each dataset were selected (supplementary materials [Supplementary-material supplementary-material-1], [Supplementary-material supplementary-material-1], and [Supplementary-material supplementary-material-1]). Finally, an online tool (http://bioinformatics.psb.ugent.be/webtools/Venn/) was used to identify the overlapping CpGs among each of the 1,000 top CpGs of GSE73003, GSE37988, and GSE57958 (Supplementary [Supplementary-material supplementary-material-1]).

After identifying the most significant differential CpGs between HCC tumors and nontumor tissues, we next verified these CpGs among HCC patients from TCGA (https://cancergenome.nih.gov/). DNA methylation profiling data of 377 HCC patients were downloaded from TCGA. The methylation profiling data were assessed on the GPL13534 platform (Illumina Human Methylation 450 BeadChip), and the methylation level was presented as a *β* value, which was calculated as the ratio of the intensity of the methylated bead type to the combined locus intensity and ranged from 0 to 1. Subsequently, clinical characteristics including sex, age, BMI, APF, cirrhosis, Child-Pugh stage, adjacent hepatic tissue inflammation, tumor histological grade, surgical margin resection status, AJCC TNM stage, and overall survival (OS) time were also downloaded. Six of 377 HCC patients were excluded because of the absence of OS data. In total, 371 HCC patients with available methylation data and clinical parameters were included in the present study. The clinical parameters of the HCC patients are summarized in Table 1.

### 2.3. Identification and Selection of HCC Prognosis-Related CpGs

Three hundred seventy-one HCC patients were randomly divided into a development cohort (*N* = 263) and a training cohort (*N* = 108) with an allocation of 7 : 3 performed by R software. The development cohort was used to identify key HCC prognosis-related CpGs and develop the MSH. Two different algorithms, least absolute shrinkage and selection operator (LASSO) analysis [15] and robust likelihood-based survival analysis [16, 17], were used to select the most significant methylation markers. Overlapping CpGs between the two selection methods were finally identified as the HCC prognosis-related CpGs.

### 2.4. Development and Validation of the MSH

After the key prognosis-related CpGs were selected, we next used them to develop the MSH by multivariable Cox regression analysis. With this model, a risk score for each HCC patient was calculated. HCC patients were further classified into a high-risk group and a low-risk group based on the cut-off value of the median risk score. The OS difference between the high-risk patients and low-risk patients was analyzed by Kaplan-Meier analysis. Then, the MSH was validated in the validation cohort. Univariate and multivariate Cox regression analyses were used to further assess the association of MSH with OS in the development and validation cohorts. Furthermore, stratified analysis was also performed to explore the influence of other major clinicopathologic factors (including AFP, cirrhosis, Child-Pugh stage, tumor histologic grade, and AJCC TNM stage) on the prognostic value of MSH in the total cohort by Kaplan–Meier analysis.

### 2.5. Establishment of a Time-Dependent Receiver Operating Characteristic (ROC) Curve and an MSH-Based Nomogram

To further assess the predictive accuracy and sensitivity of the MSH, time-dependent ROC analysis was performed with HCC patients in the total cohort. The areas under the ROC curve (AUCs) of the MSH for predicting 1-year, 3-year, and 5-year OS were calculated and used for comparisons with other models. Moreover, to make MSH more clinically applicable, an MSH-based nomogram was also developed.

### 2.6. *Gene Ontology* (GO) and *Kyoto Encyclopedia of Genes and Genomes* (*KEGG*) Analysis of the MSH

To explore the biological function and pathways of the MSH, *GO* and *KEGG* analyses were conducted. First, the 50 most frequently altered genes related to these 14 genes were downloaded from *cBioPortal* (http://www.cbioportal.org). The biological function of these 50 genes and the 14 genes were then analyzed by *GO* and *KEGG* in the Database for Annotation, Visualization, and Integrated Discovery (*DAVID*) (https://david.ncifcrf.gov/summary.jsp). The detailed method was described in our previous study [18].

### 2.7. Statistical Analysis

Statistical analysis was performed with R software (R version 3.5.1) and GraphPad Prism software (version 6). Univariate and multivariate Cox regression analyses were performed with the survival and survminer packages. The robust likelihood-based survival analysis was performed with the survivalROC and rbsurv packages, and the LASSO analysis was conducted with the glmnet and survival packages. Time-dependent ROC analysis was performed with the ROCR and rms packages. The nomogram was constructed with the rms and survival packages and was evaluated by the concordance index and calibration plots. Kaplan–Meier analysis was performed with GraphPad Prism software and was compared with the log-rank test. *P* < 0.05 was considered statistically significant.

## 3. Results

### 3.1. Basic Characteristics of the 371 HCC Patients

**Figure 1:**
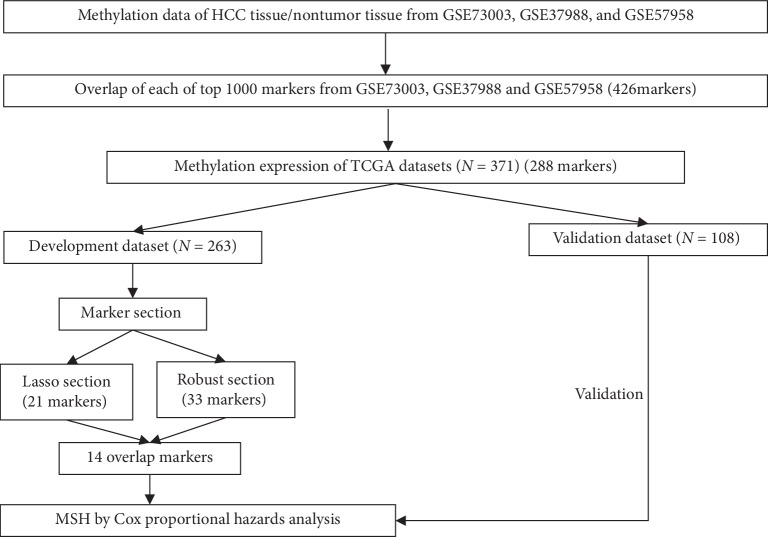
The flowchart of the development and validation of the methylation-based signature for HCC (MSH). LASSO: least absolute shrinkage and selection operator.

The flowchart of the present study is shown in Figure 1, and the basic characteristics of the 371 HCC patients are summarized in Table 1. Of the 371 HCC patients, 132 patients (35.6%) died, and 178 patients (25.2%) developed recurrence. The median OS time was 19.78 months (ranging from 0.03 to 120.73 months).

### 3.2. Selection of Key HCC Prognosis-Related CpGs

**Figure 2:**
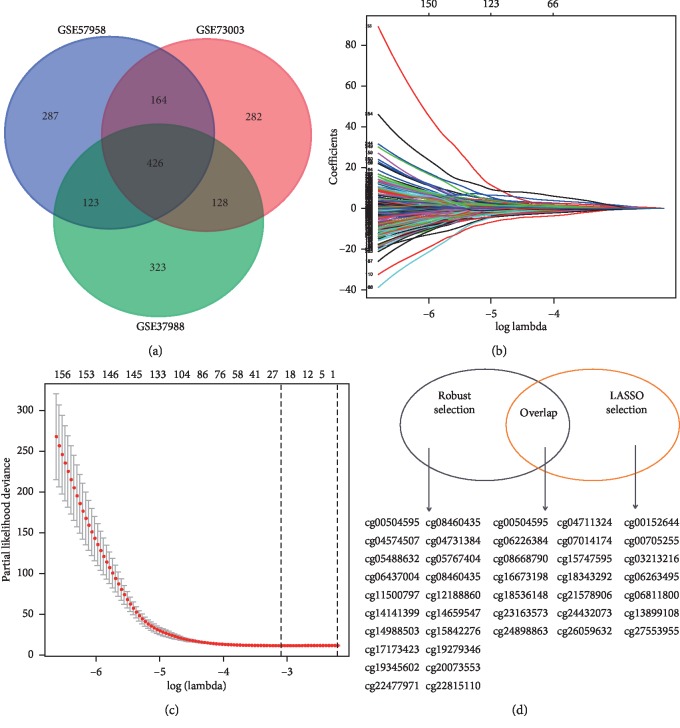
Selection of key prognosis-associated CpGs. (a) Overlap of each of the top 1000 differential CpGs from GSE73003, GSE37988, and GSE57958; (b) and (c) 21 prognosis-associated CpGs were identified by LASSO analysis in the development cohort; (d) 14 overlap CpGs were selected by robust likelihood-based survival analysis and LASSO analysis in the development cohort.

Based on the primary filter criteria, 426 differential CpGs between primary HCC tumors and the corresponding nontumor tissue were identified (Figure 2(a)). Then, we validated these selected CpGs in the HCC patients in TCGA and identified 288 CpGs that were detected by two different DNA methylation detection methods (Supplementary [Supplementary-material supplementary-material-1]). Next, LASSO analysis was used to obtain a set of 21 CpGs (Figures 2(b)–2(d)). A robust likelihood-based survival analysis was also performed and identified a set of 33 CpGs (Table 2). There were 14 overlapping CpGs between the two selection methods (Figure 2(d)), which corresponded to cg00504595 (TNF receptor superfamily member 19, TNFRSF19), cg04711324 (ras-like without CAAX2, RIT2), cg06226384 (calcium voltage-gated channel auxiliary subunit gamma 5, CACNG5), cg07014174 (keratin-associated protein 11-1, KRTAP11-1), cg08668790 (zinc finger protein 154, ZNF154), cg15747595 (TSPY-like 5, TSPYL5), cg16673198 (copine 4, CPNE4), cg18343292 (membrane spanning 4-domains A7, MS4A7), cg18536148 (T-box 4, TBX4), cg21578906 (solute carrier family 5 member 4, SLC5A4), cg23163573 (sulfotransferase family 1C member 2, SULT1C2), cg24432073 (cyclin dependent kinase-like 2, CDKL2), cg24898863 (S100 calcium-binding protein A8, S100A8), and cg26059632 (small proline rich protein 2A, SPRR2A). With the two different algorithms, HCC prognosis-related markers were strictly selected.

### 3.3. Construction and Validation of the MSH

**Figure 3:**
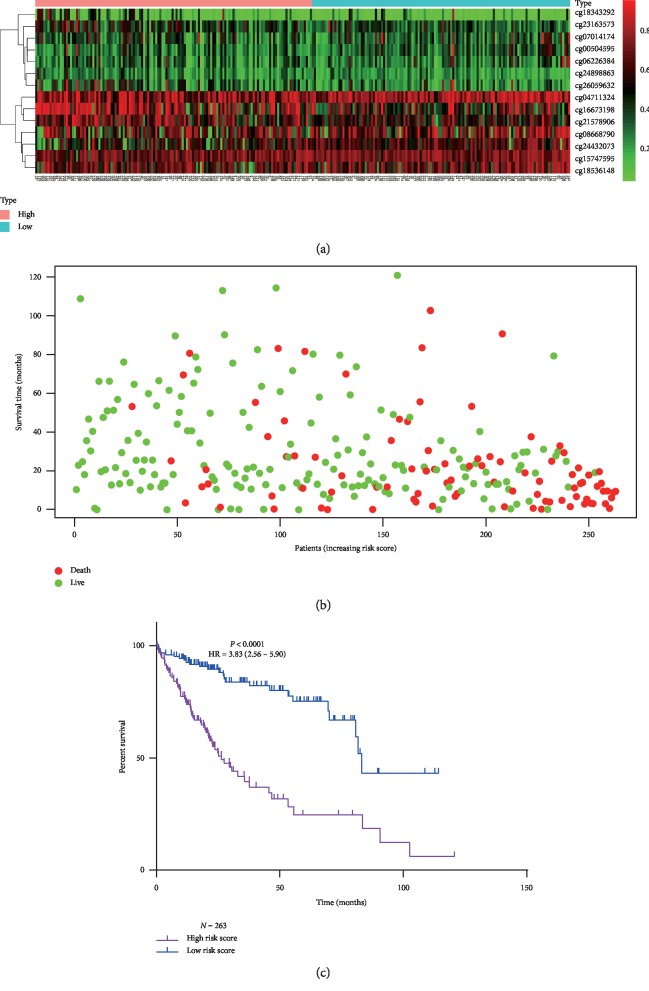
Development of 14-CpG-based signatures for HCC patients in the development cohort (*N* = 263). (a) The heat map of the methylation levels of the 14 CpGs in high-risk and low-risk patients; (b) distribution of overall status of high-risk and low-risk patients; (c) Kaplan–Meier analysis of overall survival time of high-risk and low-risk patients.

**Figure 4:**
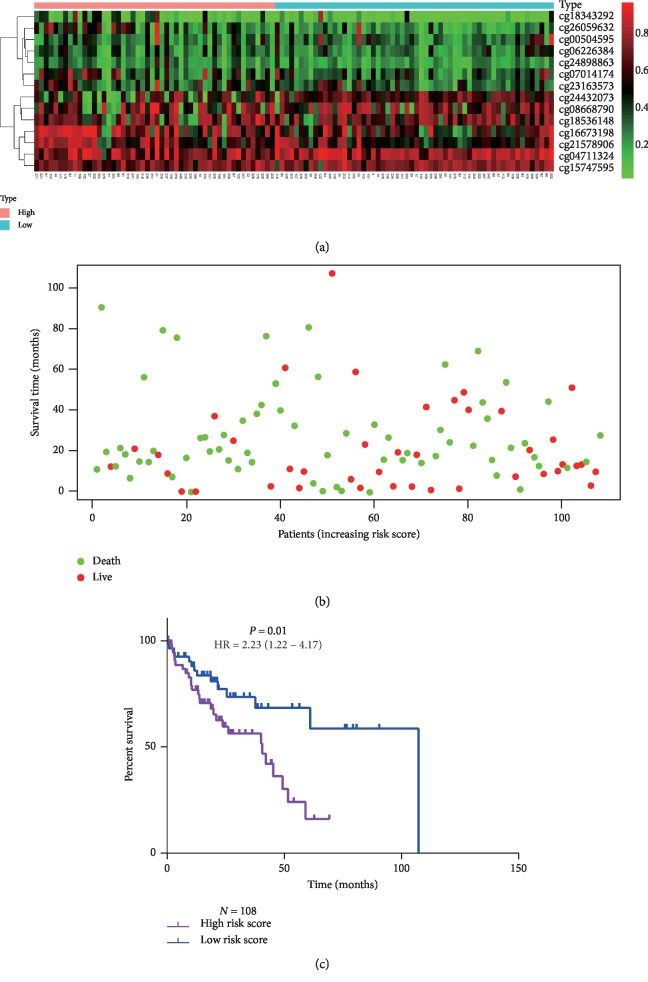
Validation of the 14-CpG-based signature among HCC patients in the validation cohort (*N* = 108). (a) The heat map of the methylation levels of the 14 CpGs in high-risk and low-risk patients; (b) distribution of overall status of high-risk and low-risk patients; (c) Kaplan–Meier analysis of overall survival time of high-risk and low-risk patients.

To comprehensively explore the association of these 14 selected CpGs with the prognosis of HCC patients, a MSH was built based on the coefficients weighted by multivariable Cox regression analysis in the development cohort (Table 3). The risk score was calculated as follows: risk score = (4.10 ∗ cg00504595) + (3.79 ∗ cg04711324) + (2.83 ∗ cg06226384) + (1.76 ∗ cg07014174) + (0.54 ∗ cg08668790) + (1.08 ∗ cg15747595) + (−2.29 ∗ cg16673198) + (−2.76 ∗ cg18343292) + (0.59 ∗ cg18536148) + (−1.93 ∗ cg21578906) + (−1.78 ∗ cg23163573) + (1.92 ∗ cg24432073) + (−2.28 ∗ cg24898863) + (−0.60 ∗ cg26059632). After the risk score for each patient in the development cohort was calculated, patients with a risk score >1.07 (median score) were assigned to the high-risk group (*N* = 132), and the other patients were assigned to the low-risk group (*N* = 131). The methylation levels of cg00504595, cg04711324, cg06226384, cg07014174, cg08668790, cg15747595, cg18536148, and cg24432073 in patients of the high-risk group tended to be higher than those in patients of the low-risk group, while the methylation levels of cg16673198, cg18343292, cg21578906, cg23163573, cg24898863, and cg26059632 tended to be lower in patients of the high-risk group (Figure 3(a)). Moreover, patients in the high-risk group had shorter OS time than those in the low-risk group (median survival time 26.35 vs 83.18 months, HR = 3.83, 95% CI: 2.56–5.90, *P* < 0.0001, Figures 3(b) and 3(c)). To assess the utility and stability of the MSH, verification analysis was performed in the validation cohort (*N* = 108). Similarly, the MSH also successfully divided 54 patients (50%) into the high-risk group and the other 54 patients into the low-risk group. The OS time of the high-risk group was lower than that of the low-risk group (40.37 vs 107.03 months, HR = 2.23, 95% CI: 1.22–4.17, *P*=0.01, Figure 4).

### 3.4. Prognostic Value of the MSH in HCC Patients

After indicating that the MSH could be used to categorize HCC patients into high-risk (poor OS) and low-risk groups (better OS), we further evaluated the prognostic value of the MSH among HCC patients. Univariate analysis showed that the MSH was significantly associated with OS in the development cohort (HR = 4.3, 95% CI: 2.691–6.871, *P* < 0.0001, Table 4) and the validation cohort (HR = 1.979, 95% CI: 1.019–3.864, *P*=0.044, Table 5). Moreover, multivariate analysis also showed that the MSH was an independent prognostic factor for OS in the two cohorts (development cohort: HR = 6.355, 95% CI: 2.524–16, *P* < 0.0001, Table 4; validation cohort, HR = 3.379, 95% CI: 1.054–10.834, *P*=0.041, Table 5).

### 3.5. Stratified Survival Analysis Based on Major Clinicopathological Factors in the Total Cohort

**Figure 5:**
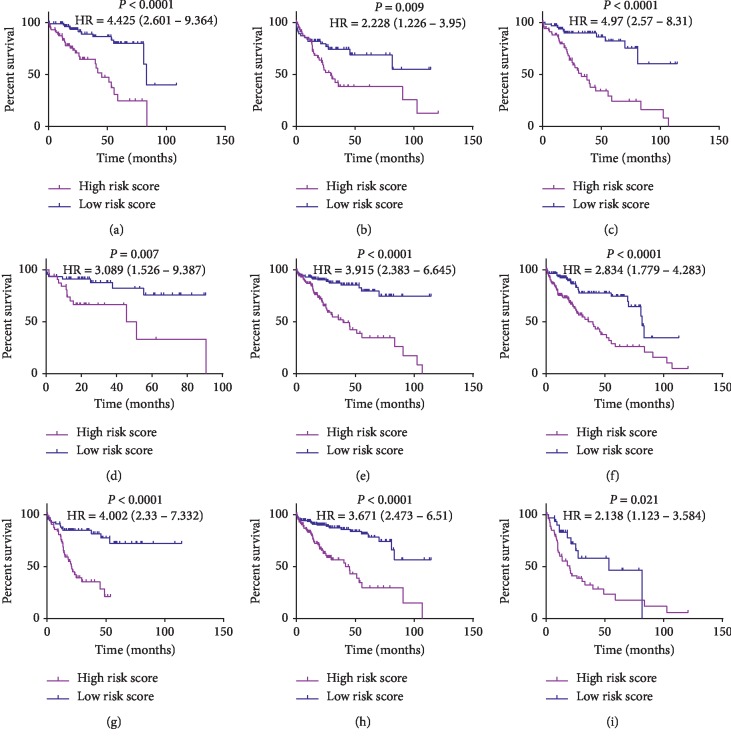
Stratified survival analysis based on major clinicopathological factors. The 14-CpG-based signature exhibited good prognostic value in different subgroups classified by AFP (a-b), cirrhosis (c-d), Child-Pugh A (e), tumor histologic grade (f-g), and AJCC stage (h-i). (a) Patients with AFP (<25) (*N* = 166). (b) Patients with AFP (>25) (*N* = 116). (c) Patients without cirrhosis (*N* = 136). (d) Patients with cirrhosis (*N* = 79). (e) Patients with Child-Pugh A (*N* = 222). (f) Patients with histologic grade G1/G2 (*N* = 233). (g) Patients with histologic grade G3/G4 (*N* = 132). (h) Patients with AJCC stage I/II (*N* = 259). (i) Patients with AJCC stage I/II (*N* = 88).

After the MSH was found to be an independent prognostic factor for the OS of HCC patients, we next performed stratified analysis to further explore the prognostic value of the MSH for patients classified by major clinicopathological factors in the total cohort. The number of patients divided into high-risk and low-risk groups and the log-rank tests are shown in Table 6. Our results indicated that the MSH still exhibited good prognostic value in different subgroups classified by AFP, cirrhosis, Child-Pugh A, tumor histologic grade, and AJCC stage (Figure 5), which, to some extent, suggested the greater reliability and general utility of the MSH.

### 3.6. Predictive Value of the MSH for the OS of HCC Patients and Comparison with Other CpG-Based Models Based on TCGA

**Figure 6:**
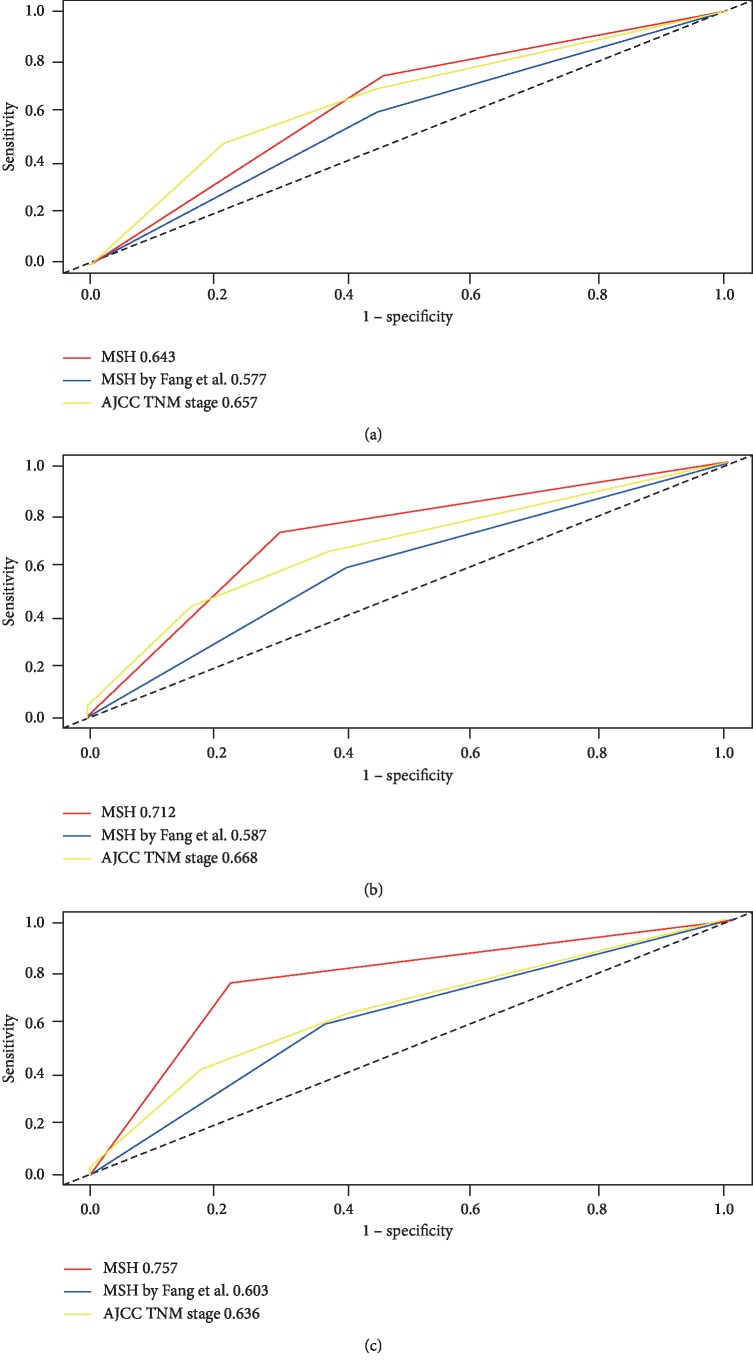
Comparison of the predictive value of the 14-CpG-based signature with AJCC TNM stage and other CpG-based signatures from TCGA. Time-dependent ROC analysis was used to evaluate the predictive value among the three signatures in predicting 1-year (a), 3-year (b), and 5-year (c) overall survival time.

Time-dependent ROC cure analysis was used to assess the predictive value of the MSH in HCC patients in the total cohort, and this analysis was used to compare the MSH to other CpG-based models based on TCGA. As shown in Figure 6, the AUCs of the MSH for predicting 1-, 3-, and 5-year OS were 0.643, 0.712, and 0.757, respectively, while the AUCs of AJCC stage, which is often used as prognostic model for HCC patients, were 0.657, 0.668, and 0.636, respectively, suggesting that the MSH exhibited a better efficiency in predicting 3- and 5-year OS.

Recently, a five-CpG-based prognostic signature was constructed by Fang et al. on the basis of HCC patients from TCGA [14], and the AUCs of the MSH developed by Fang et al. for predicting 1-, 3-, and 5-year OS were 0.577, 0.587, and 0.603, respectively. Compared to the MSH developed by Fang et al., our 14-CpG-based prognostic signature showed a favorable predictive value in predicting 1-, 3-, and 5-year OS. However, further investigation into external HCC cohorts is needed.

### 3.7. Development of an MSH-Based Nomogram for OS Prediction in HCC Patients in the Total Cohort

**Figure 7:**
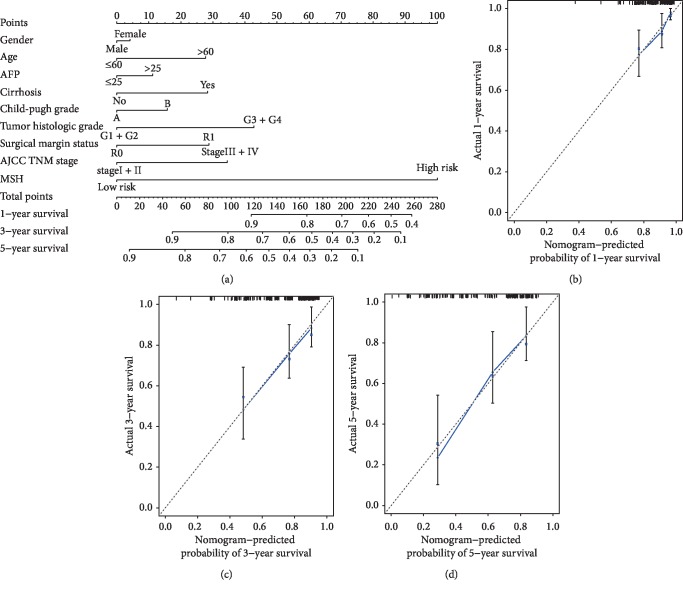
Development of an MSH-based nomogram for OS prediction in HCC patients. A nomogram, composed of sex, age, AFP, cirrhosis, Child-Pugh stage, tumor histologic grade, AJCC stage, surgical margin status, and MSH, was developed (a). Calibration curve for the MSH-based nomogram in predicting 1-year (b), 3-year (c), and 5-year (d) overall survival time.

To make MSH more clinically applicable, we developed an MSH-based nomogram to predict the 1-year, 3-year, and 5-year OS of HCC patients in the total cohort (Figure 7(a)). Clinicopathological factors, such as sex, age, AFP, cirrhosis, Child-Pugh stage, tumor histologic grade, AJCC stage, and surgical margin status, were included in the nomogram. The C-index for 1-year, 3-year, and 5-year OS prediction was 0.709, and the calibration plots also exhibited good consistency between the predicted OS and the actual OS (Figures 7(b)–7(d)), suggesting the good predictive value of our MSH-based nomogram.

### 3.8. Biological Function and Pathways of the MSH

**Figure 8:**
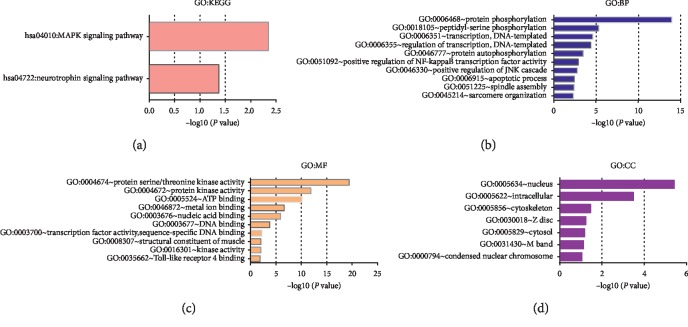
Biological function and pathways of the MSH. The biological function and pathways of the 14 genes and their 50 frequently altered neighbor genes, including KEGG pathways (a), biological processes (b), molecular functions (c), and cellular components (d).

To explore the biological function and pathways of the MSH, *GO* and *KEGG* analyses were performed. Our results showed that the MAPK signaling pathway and neurotrophin signaling pathway were affected by these 14 genes (Figure 8(a)), and as expected, these two common pathways were all reported to play important roles in the development and progression of HCC [19–21], which provided evidence for the rationality and molecular thesis of the MSH. In addition, biological processes such as GO:0006468 (protein phosphorylation), GO:0018105 (peptidyl-serine phosphorylation), and GO:0006351 (transcription, DNA-templated); molecular functions, such as GO:0004674 (protein serine/threonine kinase activity), GO:0004672 (protein kinase activity), and GO:0005524 (ATP binding); and cellular components such as GO:0005634 (nucleus), GO:0005622 (intracellular), and GO:0005856 (cytoskeleton) were also affected by these 14 genes (Figures 8(b)–8(d)).

## 4. Discussion

HCC is a highly malignant cancer with poor prognosis. It is still a great challenge to improve the clinical outcome of HCC patients because of the absence of effective prognostic biomarkers or models. In our present study, we aimed to develop and evaluate the prognostic value of methylation-based signatures for HCC patients. Fourteen candidate CpGs related to OS were identified in the development cohort by two distinct algorithms, including LASSO analysis and robust likelihood-based survival analysis. Unlike previous studies that used only one algorithm, we used two algorithms to help minimize the possibility of losing or missing important markers [22]. Subsequently, these 14 CpGs were used to develop an MSH in the development cohort and were validated in an internal validation cohort.

Our results showed that the MSH could effectively divide HCC patients into two distinct subgroups with high risk or low risk of death, suggesting the underlying clinical implications for the management of HCC patients. In addition, MSH was associated with OS and was also an independent prognostic factor for HCC patients. Moreover, stratified analysis also indicated good prognostic value in different subgroups classified by AFP, cirrhosis, Child-Pugh A, tumor histologic grade, and AJCC stage, which, to some extent, suggested the greater reliability and general utility of MSH. With the help of the MSH, high-risk HCC patients can be identified and can receive more intensive surveillance and even active adjuvant treatment to reduce recurrence and improve prognosis. Conversely, HCC patients with low risk may receive less active follow-up and even avoid adverse effects of adjuvant therapies. Therefore, MSH may be a useful method for establishing more individualized follow-up interval schedules and selecting therapeutic strategies for HCC patients after surgery.

The AJCC TNM stage is a well-known useful and common marker for predicting the prognosis of HCC. To further evaluate the predictive value of the MSH, we used time-dependent ROC analysis to compare the prediction efficacy between the MSH and AJCC stage. The prognostic predictive ability of the MSH was stable and good. The AUC for predicting 1-year, 3-year, and 5-year OS increased with increased prediction time (0.643, 0.712, and 0.757, respectively), suggesting the better accuracy of the MSH for long-time survival prediction, which is relatively important for patients at advanced stages. Compared to the AJCC stage (AUC = 0.657), the efficacy of the MSH in predicting 1-year OS was not inferior, but the efficacy of the MSH in predicting 3-year and 5-year OS was superior to that of the AJCC stage (AUC = 0.668, 0.636, respectively), indicating the advantage of MSH in predicting long-time survival. Recently, a five-CpG-based prognostic model was developed by Fang et al. on the basis of TCGA, and the AUCs of the MSH developed by Fang et al. for the prediction of 1-year, 3-year, and 5-year OS were 0.577, 0.587, and 0.603, respectively [14]. Undoubtedly, our 14-CpG-based MSH was better than the five-CpG-based MSH in predicting the OS of HCC patients. Furthermore, we built an MSH-based nomogram to make the MSH more clinically applicable. The C-index and calibration plots exhibited good consistency between the predicted OS and the actual OS, which suggested the accurate prognosis prediction of the MSH-based nomogram.

Fourteen prognosis-related CpGs correspond to TNFRSF19, RIT2, CACNG5, KRTAP11-1, ZNF154, TSPYL5, CPNE4, MS4A7, TBX4, SLC5A4, SULT1C2 CDKL2, S100A8, and SPRR2A. Among all of the CpGs, ZNF154, TSPYL5, CDKL2, and S100A8 have been reported to be associated with HCC. ZNF154, TSPYL5, and CDKL2 were found to be significantly hypermethylated and downregulated in HCC tissues compared to their methylation status in nontumor liver tissues. The methylation of TSPYL5 and CDKL2 could also be used to distinguish HCC tissues from adjacent nontumor tissues [23–27]. Consistent with the above findings, hypermethylation of ZNF154, TSPYL5, and CDKL2 was also found in HCC patients in TCGA. We also found that the methylation levels of these genes in the high-risk patients were higher than those in the low-risk patients, suggesting that ZNF154, TSPYL5, and CDKL2 may play an antitumor role in the development and progression of HCC. In contrast to ZNF154, TSPYL5, and CDKL2, significant hypomethylation of S100A8 was found in HCC tissues when compared to the methylation status of adjacent normal tissues, suggesting shorter OS and progression-free survival (PFS). In addition, overexpression of S100A8 in Huh 7 and MHCC-97H hepatoma cells resulted in increased cell proliferation, migration, and invasion [28]. Furthermore, increased expression of S100A8 and S100A9 promotes the malignant progression of HCC by activating reactive oxygen species (ROS) dependent signaling pathways and inhibiting cell death [29]. In our study, we also found hypomethylated S100A8 in patients with HCC. The methylation level of S100A8 in patients in the high-risk group was lower than that in patients in the low-risk group, indicating the important role of S100A8 in the progression of HCC, which also partly explains why HCC patients with hypomethylation of S100A8 had a shorter OS. Despite the lack of reports about the role of the other 10 genes in HCC, future characterization of these genes may provide new insights into the development and progression of HCC and the discovery of potential novel therapeutic targets for HCC.

Despite our 14-CpG-based signatures showing good performance for the prediction of the prognosis of HCC patients, several limitations of this study should be noted. First, the prognostic value of the MSH was only validated in the internal cohort from TCGA. Other external cohorts with larger sample sizes are still needed to validate our model. Second, although we explored the potential biological functions and pathways of the MSH, more experiments should be conducted to justify the related mechanisms. Finally, as noninvasive “liquid biopsy” has received increasing attention with the potential to revolutionize the diagnosis and treatment of cancer [30], whether these 14 CpGs could be detected in the blood of HCC patients and whether the signature based on these CpGs would still have good prognostic value will need further validation.

In conclusion, we identified 14 differential CpGs that were significantly associated with the OS of HCC patients. The MSH developed by these 14 CpGs showed greater advantage in terms of stability and accuracy in prognosis prediction compared to the predictive ability of AJCC stage and other CpG-based signatures from TCGA. The MSH-based nomogram may help clinicians establish more individualized therapeutic strategies for HCC patients after surgery.

## Figures and Tables

**Table 1:** Basic characteristics of the 371 HCC patients.

Variables	HCC patients (*N* = 371)
Gender (male/female)	251/120
Age (years, ≤60/>60)	176/195
BMI (≤25/>25/NA)	178/160/33
AFP (ng/ml, <25/>25/NA)	166/116/89
Cirrhosis (Yes/No/NA)	79/136/156
Child-Pugh stage (A/B/NA)	222/22/127
Adjacent tissue inflammation (Yes/No/NA)	118/119/134
Tumor histologic grade (G1/G2/G3/G4/NA)	55/178/120/13/5
Surgical margin status (R0/R1/NA)	326/18/27
AJCC TNM stage (stage1/II/III/IV/NA)	174/85/84/3/24

HCC: hepatocellular carcinoma, BMI: body mass index, NA: not available.

**Table 2:** Differential CpGs identified by robust likelihood-based survival analysis.

Gene ID	nloglik	AIC
cg17173423	433.13	868.26^*∗*^
cg20073553	431.63	867.26^*∗*^
cg12188860	431.46	868.92^*∗*^
cg21578906	429.97	867.94^*∗*^
cg06226384	421.27	852.53^*∗*^
cg04711324	416.91	845.82^*∗*^
cg08668790	416.01	846.03^*∗*^
cg00504595	413.38	842.76^*∗*^
cg22477971	411.09	840.17^*∗*^
cg18536148	410.47	840.94^*∗*^
cg14988503	410.17	842.34^*∗*^
cg24898863	408.49	840.98^*∗*^
cg11500797	408.09	842.19^*∗*^
cg05767404	407.33	842.65^*∗*^
cg00891278	407.13	844.26^*∗*^
cg19345602	404.52	841.04^*∗*^
cg26059632	403.2	840.39^*∗*^
cg05488632	402.41	840.82^*∗*^
cg15747595	401.03	840.07^*∗*^
cg04574507	399.42	838.84^*∗*^
cg04731384	399.12	840.23^*∗*^
cg06437004	399.11	842.23^*∗*^
cg15842276	398.82	843.63^*∗*^
cg22815110	398.21	844.43^*∗*^
cg23163573	397.5	844.99^*∗*^
cg14141399	397.5	846.99^*∗*^
cg08460435	396.33	846.66^*∗*^
cg16673198	392.47	840.93^*∗*^
cg18343292	389.42	836.83^*∗*^
cg19279346	389.18	838.35^*∗*^
cg24432073	385.75	833.49^*∗*^
cg07014174	384.54	833.09^*∗*^
cg14659547	383.51	833.01^*∗*^

**Table 3:** Basic characteristics of the 14 methylation markers and their coefficients weighted by multivariable Cox regression analysis (development dataset, *N* = 263).

Markers	Ref gene	Coefficients	Hazard ratio	95% CI	*P* value
cg00504595	TNFRSF19	4.10	60.34	6.51–559.57	<0.000
cg04711324	RIT2	3.79	44.11	5.49–354.79	<0.000
cg06226384	CACNG5	2.83	16.88	2.35–121.29	0.005
cg07014174	KRTAP11-1	1.76	5.83	0.95–35.72	0.056
cg08668790	ZNF154	0.54	1.72	0.66–4.51	0.269
cg15747595	TSPYL5	1.08	2.95	0.27–32.73	0.378
cg16673198	CPNE4	−2.29	0.10	0.03–0.39	<0.000
cg18343292	MS4A7	−2.76	0.06	0.01–0.52	0.01
cg18536148	TBX4	0.59	1.80	0.47–6.89	0.388
cg21578906	SLC5A4	−1.93	0.15	0.03–0.65	0.012
cg23163573	SULT1C2	−1.78	0.17	0.02–1.29	0.086
cg24432073	CDKL2	1.92	6.85	1.57–29.79	0.01
cg24898863	S100A8	−2.28	0.10	0.003–2.87	0.181
cg26059632	SPRR2A	−0.60	0.55	0.07–4.28	0.567

**Table 4:** Univariate analysis and multivariate analysis of clinicopathologic data and the MSH with OS in the development cohort.

Variables	Univariate analysis			Multivariate analysis		
	Hazard ratio	95% CI	*P* value	Hazard ratio	95% CI	*P* value
Gender (male vs female)	0.725	0.476–1.104	0.134			
Age (>60 vs <60)	1.278	0.842–1.273	0.25			
BMI (>25 vs <25)	0.849	0.546–1.321	0.469			
AFP (>25 vs <25)	1.609	0.955–2.713	0.074			
Cirrhosis (yes vs no)	1.013	0.544–1.886	0.967			
Child-Pugh stage (B vs A)	1.735	0.812–3.708	0.155			
Adjacent tissue inflammation (yes vs no)	1.157	0.647–2.068	0.623			
Tumor histologic grade (G3+G4 vs G1+G2)	1.069	0.696–1.64	0.762			
Surgical margin status (R1 vs R0)	1.545	0.671–3.558	0.306			
AJCC TNM stage (III + IV vs I + II)	1.725	1.093–2.723	0.019^*∗*^			
MSH (high risk vs low risk)	4.3	2.691–6.871	0.000^*∗*^	6.355	2.524–16.00	0.000^*∗*^

MSH: methylation signature for HCC.

**Table 5:** Univariate analysis and multivariate analysis of clinicopathologic data and the MSH with OS in the validation cohort.

Variables	Univariate analysis			Multivariate analysis		
	Hazard ratio	95% CI	*P* value	Hazard ratio	95% CI	*P* value
Gender (male vs female)	1.155	0.595–2.242	0.671			
Age (>60 vs <60 years)	1.165	0.619–2.191	0.636			
BMI (>25 vs <25)	0.834	0.419–1.661	0.606			
AFP (>25 vs <25)	1.576	0.712–3.486	0.262			
Cirrhosis (yes vs no)	0.561	0.202–1.559	0.267			
Child-Pugh stage (B vs A)	1.396	0.179–10.882	0.75			
Adjacent tissue inflammation (yes vs no)	1.239	0.520–2.953	0.628			
Tumor histologic grade (G3+G4 vs G1+G2)	1.115	0.576–2.159	0.747			
Surgical margin status (R1 vs R0)	1.714	0.523–5.609	0.373			
AJCC TNM stage (III + IV vs I + II)	5.709	2.906–11.217	0.000^*∗*^	8.884	2.533–31.16	0.001^*∗*^
MSH (high risk vs low risk)	1.979	1.019–3.864	0.044^*∗*^	3.379	1.054–10.834	0.041^*∗*^

MSH: methylation signature for HCC.

**Table 6:** Stratified survival analysis of the MSH based on major clinicopathological factors in the total cohort.

Variable	High risk	Low risk	HR (95% CI)	*P* value
Cirrhosis				
Yes	32	47	3.089 (1.526–9.387)	0.007^*∗*^
No	70	66	4.97 (2.576–8.31)	0.000^*∗*^
Child-Pugh stage				
A	102	120	3.915 (2.383–6.645)	0.000^*∗*^
B	12	10	2.735 (0.669–9.308)	0.182
Tumor histologic grade				
G1/G2	118	115	2.834 (1.779–4.283)	0.000^*∗*^
G3/G4	65	67	4.002 (2.33–7.386)	0.000^*∗*^
AJCC TNM stage				
Stage I/II	119	140	3.671 (2.473–6.516)	0.000^*∗*^
Stage III/IV	54	34	2.138 (1.123–3.584)	0.021^*∗*^
AFP				
≤25	74	92	4.425 (2.601–9.364)	0.000^*∗*^
>25	60	56	2.228 (1.226–3.95)	0.009^*∗*^
Surgical margin status				
R0	157	169	3.402 (2.361–5.011)	0.000^*∗*^
R1	11	7	1.775 (0.411–7.656)	0.4419

## Data Availability

All data in the study were obtained from online databases, including GEO (https://www.ncbi.nlm.nih.gov/geo/) and TCGA (https://cancergenome.nih.gov/).

## Abbreviations

HCC: Hepatocellular carcinoma
SNP: Single-nucleotide polymorphism
TCGA: The cancer genome atlas
MSH: Methylation-based signature for HCC
GEO: Gene Expression Omnibus
OS: Overall survival
LASSO: Least absolute shrinkage and selection operator
ROC: Receiver operating characteristic
AUC: Area under the ROC curve
GO: Gene ontology
KEGG: Kyoto encyclopedia of genes and genomes
DAVID: Database for annotation, visualization, and integrated discovery
TNFRSF19: TNF receptor superfamily member 19
RIT2: Ras-like without CAAX2
CACNG5: Calcium voltage-gated channel auxiliary subunit gamma 5
KRTAP11-1: Keratin-associated protein 11–1
ZNF154: Zinc finger protein 154
TSPYL5: TSPY-like 5
CPNE4: Copine 4
MS4A7: Membrane spanning 4-domains A7
TBX4: T-box 4
SLC5A4: Solute carrier family 5 member 4
SULT1C2: Sulfotransferase family 1C member 2
CDKL2: Cyclin dependent kinase-like 2
S100A8: S100 calcium-binding protein A8
SPRR2A: Small proline rich protein 2A
PFS: Progression-free survival
ROS: Reactive oxygen species.

## Supplementary Materials

Supplementary Materials Supplementary material 1: top 1000 differential CpGs between primary HCC tumors and their corresponding nontumor counterparts of GSE37988. Supplementary material 2: top 1000 differential CpGs between primary HCC tumors and their corresponding nontumor counterparts of GSE57958. Supplementary material 3: top 1000 differential CpGs between primary HCC tumors and their corresponding nontumor counterparts of GSE73003. Supplementary material 4: overlap of each top 1000 differential CpGs from GSE73003, GSE37988, and GSE57958 (426markers). Supplementary material 5: validated differential CpGs of GSE73003, GSE37988, and GSE57958 in HCC patients of TCGA (288 markers).
