# Supplementary material for: 14-CpG-Based Signature Improves the Prognosis Prediction of Hepatocellular Carcinoma Patients
Source: Biomed Res Int. 2020 Jan 4;2020:9762067. doi: 10.1155/2020/9762067 (PMC6970499; doi:10.1155/2020/9762067)
Supplement: Supplementary Materials — Supplementary material 1: top 1000 differential CpGs between primary HCC tumors and their corresponding nontumor counterparts of GSE37988. Supplementary material 2: top 1000 differential CpGs between primary HCC tumors and their corresponding nontumor counterparts of GSE57958. Supplementary material 3: top 1000 differential CpGs between primary HCC tumors and their corresponding nontumor counterparts of GSE73003. Supplementary material 4: overlap of each top 1000 differential CpGs from GSE73003, GSE37988, and GSE57958 (426markers). Supplementary material 5: validated differential CpGs of GSE73003, GSE37988, and GSE57958 in HCC patients of TCGA (288 markers). [file 9762067.f1.zip › 9762067.f1/supplementary material 1.docx]

**Top 1000 differential CpGs between primary HCC tumors and their corresponding non-tumor counterparts of GSE37988**

| cg09099744 |
| --- |
| cg12680609 |
| cg10895543 |
| cg21790626 |
| cg14988503 |
| cg24432073 |
| cg12840719 |
| cg08668790 |
| cg14310034 |
| cg03975694 |
| cg04786857 |
| cg11653709 |
| cg07752420 |
| cg24816455 |
| cg11009596 |
| cg05684891 |
| cg21643045 |
| cg09260089 |
| cg25564800 |
| cg25462303 |
| cg21460081 |
| cg04574507 |
| cg23865698 |
| cg08886154 |
| cg01772980 |
| cg24169915 |
| cg12200412 |
| cg25340403 |
| cg22113807 |
| cg03602500 |
| cg23684521 |
| cg15746620 |
| cg17718302 |
| cg26775866 |
| cg06914598 |
| cg11314684 |
| cg24304714 |
| cg10198932 |
| cg15952487 |
| cg12348970 |
| cg12891678 |
| cg17982102 |
| cg12014417 |
| cg18780284 |
| cg09120035 |
| cg16431978 |
| cg23018448 |
| cg04508649 |
| cg24352499 |
| cg11935147 |
| cg06101324 |
| cg25856811 |
| cg15868302 |
| cg08468689 |
| cg07711097 |
| cg10127415 |
| cg12874092 |
| cg04600618 |
| cg27409364 |
| cg01076838 |
| cg18536148 |
| cg04505023 |
| cg12315311 |
| cg21578906 |
| cg03243946 |
| cg05840553 |
| cg13897627 |
| cg16617137 |
| cg15747595 |
| cg25612480 |
| cg18416881 |
| cg04000821 |
| cg03872376 |
| cg06353345 |
| cg24765446 |
| cg18343292 |
| cg02909790 |
| cg01808508 |
| cg25119415 |
| cg27018070 |
| cg02764897 |
| cg07014174 |
| cg08970694 |
| cg05159188 |
| cg19787037 |
| cg24607535 |
| cg13615963 |
| cg25259754 |
| cg14911395 |
| cg15552238 |
| cg23163573 |
| cg04329382 |
| cg20649991 |
| cg15669228 |
| cg11377136 |
| cg25182523 |
| cg06392096 |
| cg23595927 |
| cg15821095 |
| cg06784466 |
| cg08260959 |
| cg17301902 |
| cg16122592 |
| cg07947016 |
| cg11959435 |
| cg00891278 |
| cg09868882 |
| cg25082710 |
| cg04995095 |
| cg18129786 |
| cg26799474 |
| cg00319692 |
| cg17738194 |
| cg13760253 |
| cg01144251 |
| cg14415300 |
| cg20217872 |
| cg21831174 |
| cg25391023 |
| cg11591325 |
| cg27389185 |
| cg12949760 |
| cg08441806 |
| cg24898863 |
| cg13323752 |
| cg02311163 |
| cg19996355 |
| cg10575735 |
| cg15092802 |
| cg27420123 |
| cg17657618 |
| cg02347487 |
| cg13993218 |
| cg14776962 |
| cg05767404 |
| cg22268164 |
| cg09936561 |
| cg02148642 |
| cg04322134 |
| cg04057858 |
| cg16812893 |
| cg05521696 |
| cg00466436 |
| cg00718513 |
| cg06118312 |
| cg20312687 |
| cg18484189 |
| cg25446086 |
| cg21529807 |
| cg26164184 |
| cg14757492 |
| cg13462129 |
| cg00679556 |
| cg24377133 |
| cg18462653 |
| cg07548313 |
| cg13530039 |
| cg07022477 |
| cg23338195 |
| cg24861272 |
| cg07664183 |
| cg06437004 |
| cg26117023 |
| cg10971790 |
| cg17357062 |
| cg27513764 |
| cg11884243 |
| cg22815110 |
| cg20895028 |
| cg18984151 |
| cg20845050 |
| cg20305726 |
| cg19370451 |
| cg12108912 |
| cg24870391 |
| cg16744741 |
| cg16592658 |
| cg03014957 |
| cg20654468 |
| cg12447832 |
| cg02593766 |
| cg27120999 |
| cg11005826 |
| cg06906435 |
| cg00564163 |
| cg07378350 |
| cg11015241 |
| cg01871995 |
| cg25072962 |
| cg26207503 |
| cg01072821 |
| cg02284188 |
| cg27071517 |
| cg04837071 |
| cg14576824 |
| cg20542190 |
| cg04138756 |
| cg08539093 |
| cg19356189 |
| cg18988110 |
| cg08458487 |
| cg04386405 |
| cg03311899 |
| cg18279742 |
| cg02946850 |
| cg19779211 |
| cg26848126 |
| cg25084878 |
| cg08684473 |
| cg15811427 |
| cg25651984 |
| cg17405586 |
| cg03914397 |
| cg15149938 |
| cg01169726 |
| cg06974755 |
| cg14738823 |
| cg08458170 |
| cg13899108 |
| cg05252264 |
| cg01309152 |
| cg03544379 |
| cg10143146 |
| cg19517525 |
| cg27344326 |
| cg07545232 |
| cg24024214 |
| cg05023540 |
| cg17031773 |
| cg13351583 |
| cg10334928 |
| cg09558502 |
| cg11808874 |
| cg14076161 |
| cg06244417 |
| cg24107142 |
| cg04645843 |
| cg07608333 |
| cg04999691 |
| cg01215061 |
| cg02332073 |
| cg18183281 |
| cg06233985 |
| cg21513385 |
| cg04962134 |
| cg04103514 |
| cg03109316 |
| cg20018806 |
| cg16872071 |
| cg19226099 |
| cg17361154 |
| cg19921353 |
| cg09872616 |
| cg03515901 |
| cg17687962 |
| cg23642747 |
| cg25372195 |
| cg17886204 |
| cg10883352 |
| cg21307628 |
| cg01055695 |
| cg03818682 |
| cg18055394 |
| cg17327492 |
| cg10533434 |
| cg27043873 |
| cg24824840 |
| cg16303562 |
| cg27016494 |
| cg04721098 |
| cg25384595 |
| cg14086122 |
| cg00515905 |
| cg14236389 |
| cg22951794 |
| cg11435943 |
| cg05208878 |
| cg21785536 |
| cg15842276 |
| cg01598642 |
| cg00260778 |
| cg16514843 |
| cg12685753 |
| cg24624841 |
| cg25388528 |
| cg16953612 |
| cg19856444 |
| cg07950803 |
| cg08555657 |
| cg03941108 |
| cg14162076 |
| cg00152644 |
| cg04349727 |
| cg14062083 |
| cg06952310 |
| cg14940420 |
| cg02812142 |
| cg02497758 |
| cg21023114 |
| cg23815000 |
| cg26738880 |
| cg26829529 |
| cg10503138 |
| cg05436658 |
| cg07459489 |
| cg00481227 |
| cg00427635 |
| cg20099806 |
| cg06291867 |
| cg04439215 |
| cg19776201 |
| cg27419217 |
| cg01074640 |
| cg19554294 |
| cg02442161 |
| cg13407883 |
| cg07092725 |
| cg07973461 |
| cg17827767 |
| cg22477971 |
| cg16742703 |
| cg02947253 |
| cg02331561 |
| cg01731341 |
| cg13118849 |
| cg22253945 |
| cg18534730 |
| cg07237939 |
| cg20182358 |
| cg25336198 |
| cg12682367 |
| cg08510456 |
| cg14444710 |
| cg15408454 |
| cg16817891 |
| cg03741352 |
| cg15014458 |
| cg06255227 |
| cg17034109 |
| cg04545516 |
| cg01668126 |
| cg03022541 |
| cg00705255 |
| cg23350580 |
| cg14826683 |
| cg10766289 |
| cg22646937 |
| cg23743472 |
| cg19486673 |
| cg05807444 |
| cg02601403 |
| cg01962826 |
| cg25107903 |
| cg13928961 |
| cg12506373 |
| cg22478614 |
| cg21674595 |
| cg26312920 |
| cg08088390 |
| cg07816074 |
| cg02677802 |
| cg21554552 |
| cg00918005 |
| cg00138126 |
| cg27553955 |
| cg12150401 |
| cg05440289 |
| cg08424423 |
| cg07297178 |
| cg27299588 |
| cg18849169 |
| cg23818978 |
| cg25268283 |
| cg00504595 |
| cg25674286 |
| cg00491404 |
| cg22190114 |
| cg18766755 |
| cg09076077 |
| cg14026971 |
| cg14366598 |
| cg13792279 |
| cg26059632 |
| cg04086012 |
| cg19863740 |
| cg16899306 |
| cg22229142 |
| cg06144905 |
| cg24063382 |
| cg23984130 |
| cg24549507 |
| cg20582779 |
| cg12970081 |
| cg19795898 |
| cg15538820 |
| cg01770400 |
| cg20131968 |
| cg10671066 |
| cg15494458 |
| cg25720804 |
| cg00116838 |
| cg20070090 |
| cg06194186 |
| cg27292431 |
| cg24884084 |
| cg02784874 |
| cg20437604 |
| cg07745725 |
| cg08786003 |
| cg23493704 |
| cg19982860 |
| cg25661884 |
| cg21030400 |
| cg07706362 |
| cg13158571 |
| cg18873386 |
| cg14972271 |
| cg05414338 |
| cg14603345 |
| cg25093045 |
| cg12493906 |
| cg19345602 |
| cg04151683 |
| cg10266490 |
| cg25509184 |
| cg17928268 |
| cg12796229 |
| cg11161417 |
| cg18555440 |
| cg20080624 |
| cg07408740 |
| cg24851490 |
| cg21808053 |
| cg11554507 |
| cg03504701 |
| cg20916523 |
| cg01469547 |
| cg08840010 |
| cg09555879 |
| cg26550234 |
| cg20322977 |
| cg00949442 |
| cg15238224 |
| cg19290962 |
| cg01543654 |
| cg16998872 |
| cg24217877 |
| cg11710560 |
| cg22289115 |
| cg16542081 |
| cg00126657 |
| cg01193293 |
| cg09478478 |
| cg06256735 |
| cg24355048 |
| cg01375871 |
| cg02423618 |
| cg13758677 |
| cg19797376 |
| cg16584172 |
| cg14704941 |
| cg15016628 |
| cg05659947 |
| cg08420900 |
| cg02876062 |
| cg01868128 |
| cg10500909 |
| cg00601486 |
| cg04520391 |
| cg03609102 |
| cg26540515 |
| cg14115346 |
| cg10707565 |
| cg04041960 |
| cg02807948 |
| cg02100848 |
| cg06196379 |
| cg20579480 |
| cg25839766 |
| cg10370591 |
| cg09134726 |
| cg08999352 |
| cg08124722 |
| cg17940013 |
| cg11009736 |
| cg19144013 |
| cg20998885 |
| cg27239157 |
| cg05779068 |
| cg26390526 |
| cg21355508 |
| cg22643217 |
| cg10399228 |
| cg11846968 |
| cg16242770 |
| cg17173423 |
| cg00463848 |
| cg00895324 |
| cg14460735 |
| cg11724759 |
| cg22933847 |
| cg08097882 |
| cg04164824 |
| cg15531099 |
| cg04587910 |
| cg20676475 |
| cg26813458 |
| cg06051311 |
| cg16205058 |
| cg15670863 |
| cg02955504 |
| cg03716937 |
| cg11843304 |
| cg17560332 |
| cg10129493 |
| cg13019092 |
| cg12728629 |
| cg22199118 |
| cg01354473 |
| cg19764436 |
| cg20713492 |
| cg15606663 |
| cg24272907 |
| cg15555014 |
| cg22424444 |
| cg09841009 |
| cg07109801 |
| cg22920417 |
| cg21991396 |
| cg06812844 |
| cg01248426 |
| cg10321723 |
| cg03213216 |
| cg10080004 |
| cg22778947 |
| cg00613255 |
| cg11393848 |
| cg16016036 |
| cg06154597 |
| cg25250358 |
| cg09059945 |
| cg25944100 |
| cg22215728 |
| cg13877915 |
| cg13878010 |
| cg23776892 |
| cg01999333 |
| cg24908058 |
| cg25177139 |
| cg12339029 |
| cg19464944 |
| cg21038703 |
| cg11059341 |
| cg23110514 |
| cg00750606 |
| cg25545210 |
| cg15983005 |
| cg03127543 |
| cg14141399 |
| cg14370448 |
| cg05488632 |
| cg17501569 |
| cg11068096 |
| cg01242619 |
| cg02798801 |
| cg05126264 |
| cg16345226 |
| cg05039054 |
| cg14544583 |
| cg27495845 |
| cg23001457 |
| cg14353201 |
| cg13801416 |
| cg24080529 |
| cg12547930 |
| cg13349425 |
| cg14481339 |
| cg08878744 |
| cg22981461 |
| cg24505375 |
| cg18096388 |
| cg00661485 |
| cg24468890 |
| cg14533138 |
| cg14107638 |
| cg04484789 |
| cg03742272 |
| cg18149919 |
| cg01204985 |
| cg03330516 |
| cg21825364 |
| cg13694749 |
| cg23753610 |
| cg13139843 |
| cg12594641 |
| cg24101359 |
| cg09069593 |
| cg19623751 |
| cg11801011 |
| cg10335112 |
| cg03789934 |
| cg15739437 |
| cg01587454 |
| cg03312792 |
| cg05461841 |
| cg14127659 |
| cg23002761 |
| cg25033144 |
| cg19601328 |
| cg08995424 |
| cg02818322 |
| cg05810550 |
| cg16225091 |
| cg20073553 |
| cg17356112 |
| cg16377872 |
| cg21457147 |
| cg19216731 |
| cg04901273 |
| cg06811800 |
| cg04636557 |
| cg19713196 |
| cg15883716 |
| cg04940435 |
| cg27478659 |
| cg07221454 |
| cg06806711 |
| cg17099569 |
| cg06226384 |
| cg05627103 |
| cg06263495 |
| cg24642523 |
| cg27596068 |
| cg15787039 |
| cg07284407 |
| cg15741706 |
| cg13038560 |
| cg01680762 |
| cg04711324 |
| cg09326702 |
| cg21434954 |
| cg07409200 |
| cg18967533 |
| cg23988567 |
| cg11061975 |
| cg06501084 |
| cg21930712 |
| cg20088964 |
| cg10691387 |
| cg08023692 |
| cg09793866 |
| cg07841014 |
| cg09702010 |
| cg19388557 |
| cg24423088 |
| cg07374637 |
| cg17177699 |
| cg10848367 |
| cg21902327 |
| cg02206259 |
| cg08020808 |
| cg03100752 |
| cg04267184 |
| cg09828634 |
| cg26817573 |
| cg13297249 |
| cg08575537 |
| cg07525077 |
| cg23181133 |
| cg16639185 |
| cg09845785 |
| cg03600318 |
| cg06639544 |
| cg22471346 |
| cg11698653 |
| cg13397379 |
| cg08268099 |
| cg25484904 |
| cg12332316 |
| cg00363813 |
| cg00474004 |
| cg09340639 |
| cg03898365 |
| cg07572435 |
| cg27226949 |
| cg24964364 |
| cg23894058 |
| cg14260458 |
| cg05828624 |
| cg00546897 |
| cg17173856 |
| cg27214365 |
| cg10248727 |
| cg14284171 |
| cg12019109 |
| cg07258507 |
| cg08763351 |
| cg26090660 |
| cg25477904 |
| cg24735489 |
| cg27105123 |
| cg16869108 |
| cg02721374 |
| cg20485165 |
| cg18236477 |
| cg07265310 |
| cg06319346 |
| cg04431776 |
| cg25687894 |
| cg11128808 |
| cg20047055 |
| cg25221254 |
| cg04731384 |
| cg22647018 |
| cg19906926 |
| cg25829729 |
| cg23733753 |
| cg18457737 |
| cg13300756 |
| cg17474651 |
| cg08465862 |
| cg20311730 |
| cg18952647 |
| cg18680834 |
| cg24046474 |
| cg14659547 |
| cg25607161 |
| cg25697314 |
| cg04577715 |
| cg13577076 |
| cg25214366 |
| cg03329572 |
| cg03555203 |
| cg25098401 |
| cg23867494 |
| cg25456959 |
| cg07730329 |
| cg24276491 |
| cg07361385 |
| cg00881370 |
| cg14386312 |
| cg10576828 |
| cg19279346 |
| cg02580606 |
| cg27446233 |
| cg09617773 |
| cg08996413 |
| cg04034767 |
| cg18943383 |
| cg04983977 |
| cg13471990 |
| cg03673470 |
| cg24599942 |
| cg15928132 |
| cg18956481 |
| cg17204557 |
| cg12683641 |
| cg01987509 |
| cg11750883 |
| cg04471507 |
| cg08782122 |
| cg17607024 |
| cg26850754 |
| cg24831427 |
| cg12334759 |
| cg01138020 |
| cg07918509 |
| cg05832051 |
| cg04345908 |
| cg21577049 |
| cg10807560 |
| cg25689649 |
| cg16324018 |
| cg13798289 |
| cg19421752 |
| cg26457013 |
| cg15586352 |
| cg01546430 |
| cg10432620 |
| cg03945800 |
| cg24107665 |
| cg14153740 |
| cg13164309 |
| cg02044879 |
| cg14861570 |
| cg10210238 |
| cg15610233 |
| cg11500797 |
| cg02280309 |
| cg11762346 |
| cg01669948 |
| cg11344614 |
| cg27000831 |
| cg24812103 |
| cg07412254 |
| cg11471401 |
| cg26667975 |
| cg09966445 |
| cg13003163 |
| cg09500672 |
| cg24736099 |
| cg12397274 |
| cg05873268 |
| cg03954858 |
| cg14541950 |
| cg23663653 |
| cg15320474 |
| cg17688525 |
| cg07654934 |
| cg05799317 |
| cg23674788 |
| cg12855851 |
| cg13447818 |
| cg12718562 |
| cg00501366 |
| cg02037013 |
| cg18692273 |
| cg26063872 |
| cg10635061 |
| cg20931907 |
| cg22815214 |
| cg04956382 |
| cg06186808 |
| cg16678925 |
| cg27268486 |
| cg12188860 |
| cg16112157 |
| cg24387380 |
| cg19140639 |
| cg02910574 |
| cg26989103 |
| cg16673198 |
| cg10081899 |
| cg15417244 |
| cg06112415 |
| cg12365667 |
| cg07330329 |
| cg18201198 |
| cg06457357 |
| cg24109894 |
| cg11474811 |
| cg09847584 |
| cg13226591 |
| cg00513220 |
| cg10920765 |
| cg16614500 |
| cg14114267 |
| cg21045388 |
| cg16986846 |
| cg10135717 |
| cg15275890 |
| cg25214346 |
| cg00371195 |
| cg27090087 |
| cg27351998 |
| cg26499286 |
| cg08981777 |
| cg04828792 |
| cg02020018 |
| cg14646244 |
| cg15051063 |
| cg17205788 |
| cg05221167 |
| cg18509435 |
| cg25101056 |
| cg00280894 |
| cg15447486 |
| cg15602735 |
| cg26572597 |
| cg23192899 |
| cg26149550 |
| cg01053621 |
| cg13916742 |
| cg02978737 |
| cg07373172 |
| cg08794763 |
| cg10062065 |
| cg22861316 |
| cg12513379 |
| cg26776077 |
| cg04062391 |
| cg23413307 |
| cg25764191 |
| cg15105703 |
| cg15209169 |
| cg01605984 |
| cg18338296 |
| cg03297731 |
| cg00401678 |
| cg02936263 |
| cg02028524 |
| cg25328975 |
| cg15329483 |
| cg27234090 |
| cg18822544 |
| cg23248452 |
| cg25256723 |
| cg18239253 |
| cg20277670 |
| cg27210136 |
| cg22039287 |
| cg21230133 |
| cg23743114 |
| cg08321330 |
| cg08583049 |
| cg20017995 |
| cg05193832 |
| cg13088755 |
| cg27138018 |
| cg14519000 |
| cg12114524 |
| cg23029519 |
| cg24928687 |
| cg00136477 |
| cg10559803 |
| cg01612158 |
| cg24346637 |
| cg08999895 |
| cg13733733 |
| cg07005767 |
| cg18841952 |
| cg18564727 |
| cg21291985 |
| cg05922591 |
| cg23043245 |
| cg09947274 |
| cg19404979 |
| cg08475088 |
| cg24789424 |
| cg20119871 |
| cg01325515 |
| cg08191854 |
| cg07239938 |
| cg24751129 |
| cg14023451 |
| cg19306866 |
| cg14078518 |
| cg27488807 |
| cg01566170 |
| cg13873733 |
| cg00807586 |
| cg07572341 |
| cg26853855 |
| cg06796611 |
| cg26104469 |
| cg17229197 |
| cg11271605 |
| cg10145926 |
| cg03116740 |
| cg21306775 |
| cg07233761 |
| cg22767466 |
| cg15627025 |
| cg02250594 |
| cg02658251 |
| cg10853416 |
| cg18884741 |
| cg14187678 |
| cg03283694 |
| cg03366382 |
| cg21707816 |
| cg25483003 |
| cg12406559 |
| cg14696348 |
| cg26656135 |
| cg20950277 |
| cg13482233 |
| cg20256783 |
| cg18437633 |
| cg23391785 |
| cg22407504 |
| cg10872209 |
| cg16192029 |
| cg11173246 |
| cg13745346 |
| cg10334385 |
| cg06145357 |
| cg13588354 |
| cg08872742 |
| cg09864990 |
| cg07747336 |
| cg25778479 |
| cg02868123 |
| cg20063650 |
| cg05248781 |
| cg01637734 |
| cg17129388 |
| cg24816298 |
| cg14532417 |
| cg24926042 |
| cg05158615 |
| cg19930802 |
| cg23158811 |
| cg08460435 |
| cg03803009 |
| cg24694549 |
| cg10011623 |
| cg20227165 |
| cg15926585 |
| cg27345534 |
