# Supplementary material for: 14-CpG-Based Signature Improves the Prognosis Prediction of Hepatocellular Carcinoma Patients
Source: Biomed Res Int. 2020 Jan 4;2020:9762067. doi: 10.1155/2020/9762067 (PMC6970499; doi:10.1155/2020/9762067)
Supplement: Supplementary Materials — Supplementary material 1: top 1000 differential CpGs between primary HCC tumors and their corresponding nontumor counterparts of GSE37988. Supplementary material 2: top 1000 differential CpGs between primary HCC tumors and their corresponding nontumor counterparts of GSE57958. Supplementary material 3: top 1000 differential CpGs between primary HCC tumors and their corresponding nontumor counterparts of GSE73003. Supplementary material 4: overlap of each top 1000 differential CpGs from GSE73003, GSE37988, and GSE57958 (426markers). Supplementary material 5: validated differential CpGs of GSE73003, GSE37988, and GSE57958 in HCC patients of TCGA (288 markers). [file 9762067.f1.zip › 9762067.f1/supplementary material 2.docx]

**Top 1000 differential CpGs between primary HCC tumors and their corresponding non-tumor counterparts of GSE57958**

| cg04786857 |
| --- |
| cg05023540 |
| cg09120035 |
| cg11377136 |
| cg15747595 |
| cg24432073 |
| cg00949442 |
| cg04809136 |
| cg05684891 |
| cg21790626 |
| cg09099744 |
| cg25856811 |
| cg05767404 |
| cg00891278 |
| cg25119415 |
| cg04962134 |
| cg12680609 |
| cg08886154 |
| cg12891678 |
| cg05488632 |
| cg02677802 |
| cg18572014 |
| cg06437004 |
| cg11935147 |
| cg21643045 |
| cg27409364 |
| cg04138756 |
| cg16812893 |
| cg08668790 |
| cg19553463 |
| cg21825027 |
| cg06101324 |
| cg12493906 |
| cg04995095 |
| cg13897627 |
| cg07950803 |
| cg18780284 |
| cg03602500 |
| cg00495491 |
| cg15657668 |
| cg15602809 |
| cg25002911 |
| cg03914397 |
| cg21284731 |
| cg08555657 |
| cg19787037 |
| cg04600618 |
| cg23391785 |
| cg10895543 |
| cg12971694 |
| cg02655204 |
| cg07545232 |
| cg03109316 |
| cg23865698 |
| cg27071517 |
| cg19982860 |
| cg25612480 |
| cg00705255 |
| cg10853416 |
| cg19418515 |
| cg25259754 |
| cg16122592 |
| cg03504701 |
| cg23410113 |
| cg02284188 |
| cg24027342 |
| cg10910775 |
| cg15014458 |
| cg03960217 |
| cg08878744 |
| cg03705396 |
| cg14162076 |
| cg02784874 |
| cg05480532 |
| cg17738194 |
| cg18343292 |
| cg03741352 |
| cg04574507 |
| cg00895324 |
| cg23350580 |
| cg14533138 |
| cg07265310 |
| cg03826976 |
| cg15531099 |
| cg15552238 |
| cg04505023 |
| cg01962826 |
| cg07947016 |
| cg26813458 |
| cg26986815 |
| cg24169915 |
| cg16431978 |
| cg14399656 |
| cg20095587 |
| cg25720804 |
| cg16744741 |
| cg07752420 |
| cg16303562 |
| cg00503840 |
| cg15408454 |
| cg24870391 |
| cg18967533 |
| cg15787039 |
| cg06906435 |
| cg25033144 |
| cg10370591 |
| cg00152644 |
| cg03975694 |
| cg17703554 |
| cg05126264 |
| cg00601486 |
| cg25545210 |
| cg04774694 |
| cg06428055 |
| cg01074640 |
| cg07841014 |
| cg27478659 |
| cg00138126 |
| cg15983005 |
| cg16514843 |
| cg21349901 |
| cg22478614 |
| cg19226099 |
| cg17740305 |
| cg06196379 |
| cg06392096 |
| cg09555879 |
| cg12150401 |
| cg22861316 |
| cg01668126 |
| cg22496652 |
| cg09260089 |
| cg13397379 |
| cg02813121 |
| cg25839766 |
| cg04958389 |
| cg25462303 |
| cg01772980 |
| cg13300756 |
| cg10129493 |
| cg03561565 |
| cg08970694 |
| cg02148642 |
| cg10335112 |
| cg11102782 |
| cg03022541 |
| cg23563234 |
| cg17327492 |
| cg27214365 |
| cg17827767 |
| cg25384595 |
| cg12949760 |
| cg10127415 |
| cg04726446 |
| cg14988503 |
| cg13928961 |
| cg17718302 |
| cg10807560 |
| cg04901273 |
| cg23674788 |
| cg17357062 |
| cg25093045 |
| cg02601403 |
| cg22375192 |
| cg22984277 |
| cg00958560 |
| cg12348970 |
| cg14236389 |
| cg23001457 |
| cg15627025 |
| cg26087862 |
| cg11161417 |
| cg06244417 |
| cg02787991 |
| cg06914598 |
| cg13758677 |
| cg16998872 |
| cg05675373 |
| cg27344326 |
| cg02812142 |
| cg19623751 |
| cg21230133 |
| cg00213044 |
| cg02658251 |
| cg12339029 |
| cg18766755 |
| cg22951794 |
| cg11009736 |
| cg12078929 |
| cg18555440 |
| cg03070194 |
| cg14911395 |
| cg27420123 |
| cg09841009 |
| cg27504299 |
| cg06353345 |
| cg12682367 |
| cg10377274 |
| cg23413307 |
| cg13102585 |
| cg26829529 |
| cg24110050 |
| cg01215061 |
| cg02423618 |
| cg13407883 |
| cg11653709 |
| cg07258507 |
| cg25107903 |
| cg00280894 |
| cg14757296 |
| cg21991396 |
| cg16192029 |
| cg23293787 |
| cg00750606 |
| cg03782453 |
| cg14826683 |
| cg17281600 |
| cg13118849 |
| cg26312920 |
| cg17928268 |
| cg22039846 |
| cg12188860 |
| cg00501366 |
| cg24949488 |
| cg07297178 |
| cg24765446 |
| cg19140639 |
| cg15092802 |
| cg08983760 |
| cg04711324 |
| cg25608041 |
| cg08321346 |
| cg25072962 |
| cg23743472 |
| cg03014957 |
| cg20131968 |
| cg12014417 |
| cg11052143 |
| cg24861272 |
| cg03872376 |
| cg21243096 |
| cg20616414 |
| cg14659547 |
| cg26776077 |
| cg23458892 |
| cg02755525 |
| cg11015241 |
| cg08786003 |
| cg03818682 |
| cg05037688 |
| cg08872742 |
| cg04431776 |
| cg10235817 |
| cg02332073 |
| cg14141399 |
| cg06291867 |
| cg08766149 |
| cg18140857 |
| cg15602735 |
| cg12200412 |
| cg17405586 |
| cg09448880 |
| cg08611714 |
| cg06319346 |
| cg08539093 |
| cg20070090 |
| cg15375239 |
| cg02876062 |
| cg01144251 |
| cg17200465 |
| cg20649991 |
| cg20312687 |
| cg10556064 |
| cg13792279 |
| cg20256783 |
| cg17483510 |
| cg08214957 |
| cg02868123 |
| cg24063382 |
| cg14062083 |
| cg24423088 |
| cg02593766 |
| cg01868128 |
| cg08996413 |
| cg15329483 |
| cg04322134 |
| cg07092725 |
| cg11710560 |
| cg18815943 |
| cg00918005 |
| cg14366598 |
| cg10938286 |
| cg24355048 |
| cg14541311 |
| cg05440289 |
| cg12998491 |
| cg12108912 |
| cg22898761 |
| cg06572160 |
| cg17173423 |
| cg12315311 |
| cg27553955 |
| cg10503138 |
| cg21825364 |
| cg08268099 |
| cg12840719 |
| cg24824840 |
| cg21801378 |
| cg25942450 |
| cg17267907 |
| cg09610963 |
| cg01731341 |
| cg04439215 |
| cg24660086 |
| cg07038400 |
| cg09422355 |
| cg20311730 |
| cg14310034 |
| cg20227165 |
| cg21269934 |
| cg08441806 |
| cg17560332 |
| cg09076077 |
| cg08684473 |
| cg23753610 |
| cg07703401 |
| cg25651984 |
| cg12718562 |
| cg16791686 |
| cg08458170 |
| cg14696820 |
| cg12728629 |
| cg03544379 |
| cg13993218 |
| cg20692569 |
| cg24107142 |
| cg01913908 |
| cg13694749 |
| cg23163573 |
| cg21902327 |
| cg11061975 |
| cg25082710 |
| cg16076328 |
| cg08590939 |
| cg26059632 |
| cg05190718 |
| cg27513764 |
| cg20073553 |
| cg03941108 |
| cg18534730 |
| cg23984130 |
| cg17687962 |
| cg23776892 |
| cg22988566 |
| cg01598642 |
| cg22892110 |
| cg27120999 |
| cg03803009 |
| cg25306170 |
| cg10523494 |
| cg17356112 |
| cg22477971 |
| cg18521771 |
| cg26216632 |
| cg12114524 |
| cg10575735 |
| cg27043873 |
| cg17271365 |
| cg14917512 |
| cg11959435 |
| cg19370451 |
| cg13733733 |
| cg25098401 |
| cg02909790 |
| cg07747336 |
| cg03902905 |
| cg25336198 |
| cg20485165 |
| cg13350783 |
| cg04721098 |
| cg15415545 |
| cg07745725 |
| cg20200335 |
| cg25890048 |
| cg24735489 |
| cg01055695 |
| cg17886204 |
| cg10521852 |
| cg11698653 |
| cg26563737 |
| cg15606663 |
| cg07434382 |
| cg24252809 |
| cg07017706 |
| cg03805684 |
| cg14456683 |
| cg21988465 |
| cg26090660 |
| cg08260959 |
| cg22767466 |
| cg07459489 |
| cg13181284 |
| cg00463848 |
| cg04086012 |
| cg10248727 |
| cg16016036 |
| cg08460435 |
| cg02721374 |
| cg09864990 |
| cg21256656 |
| cg26117023 |
| cg18020749 |
| cg14087150 |
| cg10883621 |
| cg01193293 |
| cg18691434 |
| cg06748315 |
| cg00512031 |
| cg00679556 |
| cg22680204 |
| cg19216731 |
| cg18221897 |
| cg25176823 |
| cg20444256 |
| cg08124722 |
| cg07711097 |
| cg01169726 |
| cg12334759 |
| cg20305726 |
| cg19464944 |
| cg11435943 |
| cg04034767 |
| cg01469547 |
| cg21023114 |
| cg10691387 |
| cg22268164 |
| cg15952487 |
| cg26507477 |
| cg24816455 |
| cg15944856 |
| cg16225091 |
| cg16970232 |
| cg18536148 |
| cg20119871 |
| cg17725968 |
| cg19996355 |
| cg01227519 |
| cg18841952 |
| cg01808508 |
| cg04828792 |
| cg03860768 |
| cg04000821 |
| cg15586352 |
| cg07973461 |
| cg10246520 |
| cg13246269 |
| cg20277670 |
| cg03742272 |
| cg12588301 |
| cg06811800 |
| cg07259382 |
| cg20520725 |
| cg11237817 |
| cg11068096 |
| cg25477904 |
| cg25764191 |
| cg16778903 |
| cg23128056 |
| cg05208878 |
| cg01564343 |
| cg13164309 |
| cg05832051 |
| cg02818322 |
| cg15538820 |
| cg21663722 |
| cg02780988 |
| cg10751811 |
| cg25802093 |
| cg20018806 |
| cg10125195 |
| cg13745346 |
| cg07654934 |
| cg07374637 |
| cg20047055 |
| cg25455753 |
| cg17484237 |
| cg21663122 |
| cg14561282 |
| cg04267184 |
| cg15821095 |
| cg19863740 |
| cg03329572 |
| cg08097882 |
| cg11474811 |
| cg16272420 |
| cg00891541 |
| cg01560871 |
| cg21640749 |
| cg17264618 |
| cg26164184 |
| cg18081258 |
| cg19241311 |
| cg20099806 |
| cg14153740 |
| cg09069593 |
| cg00661485 |
| cg01295203 |
| cg04833845 |
| cg08321330 |
| cg05873268 |
| cg01587454 |
| cg04057858 |
| cg15737319 |
| cg05158615 |
| cg06324671 |
| cg12727374 |
| cg13916742 |
| cg22269180 |
| cg08822227 |
| cg05521696 |
| cg21789545 |
| cg19421752 |
| cg19290962 |
| cg26738880 |
| cg04645843 |
| cg03958979 |
| cg03312792 |
| cg18003135 |
| cg26062856 |
| cg22762309 |
| cg12970081 |
| cg08424423 |
| cg15520279 |
| cg24304714 |
| cg03716937 |
| cg12878228 |
| cg15815843 |
| cg04920951 |
| cg12833011 |
| cg03029616 |
| cg08981777 |
| cg15403517 |
| cg00546897 |
| cg04618528 |
| cg06799664 |
| cg09053680 |
| cg03213216 |
| cg11084611 |
| cg21003606 |
| cg24642468 |
| cg18564727 |
| cg12281657 |
| cg02311163 |
| cg15516226 |
| cg11750883 |
| cg25943276 |
| cg07548313 |
| cg04645174 |
| cg21065959 |
| cg06952310 |
| cg06226384 |
| cg13349425 |
| cg09174741 |
| cg12582965 |
| cg02955988 |
| cg23642747 |
| cg24908058 |
| cg18986165 |
| cg14532417 |
| cg20769842 |
| cg20856834 |
| cg00755043 |
| cg21414251 |
| cg02347487 |
| cg14826456 |
| cg22045288 |
| cg12547930 |
| cg25983380 |
| cg06639544 |
| cg04345908 |
| cg21023001 |
| cg02064402 |
| cg08742106 |
| cg02442161 |
| cg15670863 |
| cg07572435 |
| cg08430598 |
| cg00613255 |
| cg01532771 |
| cg00187686 |
| cg25372195 |
| cg21505334 |
| cg07014174 |
| cg23566335 |
| cg20931907 |
| cg25340403 |
| cg07136161 |
| cg15945602 |
| cg07940804 |
| cg23663653 |
| cg20994561 |
| cg27320127 |
| cg00188348 |
| cg19751300 |
| cg25737664 |
| cg26572597 |
| cg16029760 |
| cg24654350 |
| cg07022477 |
| cg27305303 |
| cg19642007 |
| cg23815000 |
| cg10883352 |
| cg21163415 |
| cg11843304 |
| cg27016494 |
| cg03020951 |
| cg01602596 |
| cg23988567 |
| cg16617137 |
| cg11500797 |
| cg19372178 |
| cg05779068 |
| cg11618577 |
| cg21518208 |
| cg01309152 |
| cg02992596 |
| cg19356189 |
| cg08214029 |
| cg11234457 |
| cg14444710 |
| cg04132607 |
| cg13621440 |
| cg01568736 |
| cg00627233 |
| cg02620013 |
| cg00746981 |
| cg06377278 |
| cg24926780 |
| cg14704941 |
| cg21578906 |
| cg24736099 |
| cg17964955 |
| cg14415300 |
| cg19486673 |
| cg19930802 |
| cg26644395 |
| cg15147516 |
| cg01796228 |
| cg07864297 |
| cg24352499 |
| cg10159529 |
| cg15741706 |
| cg23507131 |
| cg02982734 |
| cg05364569 |
| cg11554507 |
| cg21542793 |
| cg22218909 |
| cg25900806 |
| cg18873386 |
| cg10648113 |
| cg06256735 |
| cg03283694 |
| cg16592658 |
| cg12683641 |
| cg06791867 |
| cg15028436 |
| cg05112299 |
| cg00214794 |
| cg17696091 |
| cg24995381 |
| cg19306866 |
| cg01637734 |
| cg19258973 |
| cg08782122 |
| cg15842276 |
| cg03294557 |
| cg21434954 |
| cg22642718 |
| cg07005767 |
| cg16678925 |
| cg10707565 |
| cg09617773 |
| cg07717632 |
| cg15669228 |
| cg08999895 |
| cg18849169 |
| cg18901980 |
| cg07426848 |
| cg05252264 |
| cg06489008 |
| cg08020808 |
| cg20311501 |
| cg18741908 |
| cg18342279 |
| cg17965019 |
| cg27456885 |
| cg23595927 |
| cg16411152 |
| cg13434203 |
| cg22424444 |
| cg12954718 |
| cg08583049 |
| cg06255227 |
| cg11075556 |
| cg01078689 |
| cg09786257 |
| cg07922606 |
| cg02251134 |
| cg04731384 |
| cg03330516 |
| cg02580606 |
| cg05800321 |
| cg27429194 |
| cg08475088 |
| cg09276978 |
| cg10500909 |
| cg17474651 |
| cg25214366 |
| cg09447105 |
| cg06123346 |
| cg13410437 |
| cg22346765 |
| cg22646937 |
| cg22190114 |
| cg00718513 |
| cg20609368 |
| cg19283196 |
| cg03555203 |
| cg23338195 |
| cg22228134 |
| cg04041960 |
| cg19345602 |
| cg26063872 |
| cg23812886 |
| cg06132342 |
| cg10576828 |
| cg14284171 |
| cg21930712 |
| cg13297249 |
| cg11846968 |
| cg17036737 |
| cg27105123 |
| cg22495124 |
| cg05056120 |
| cg11158374 |
| cg24024214 |
| cg11983245 |
| cg15746620 |
| cg09847584 |
| cg13802966 |
| cg16953612 |
| cg20909686 |
| cg04448487 |
| cg13519373 |
| cg09390792 |
| cg03616357 |
| cg10848367 |
| cg04484789 |
| cg24875415 |
| cg21631409 |
| cg11465372 |
| cg02168291 |
| cg00727947 |
| cg17229197 |
| cg14869028 |
| cg16306115 |
| cg20085077 |
| cg27394486 |
| cg12038710 |
| cg20022541 |
| cg16689634 |
| cg19132372 |
| cg11484872 |
| cg14940420 |
| cg25383242 |
| cg07284407 |
| cg01375871 |
| cg07432969 |
| cg16158681 |
| cg26424956 |
| cg17205788 |
| cg19856444 |
| cg17657618 |
| cg06621358 |
| cg11520395 |
| cg05047411 |
| cg15905124 |
| cg18638581 |
| cg07126559 |
| cg02477931 |
| cg24929737 |
| cg07409200 |
| cg05248781 |
| cg13471990 |
| cg24300924 |
| cg06432655 |
| cg10604168 |
| cg14757492 |
| cg02930996 |
| cg24652919 |
| cg07525077 |
| cg27626424 |
| cg04254916 |
| cg09580336 |
| cg14535518 |
| cg11128808 |
| cg05659947 |
| cg06263495 |
| cg20542190 |
| cg02659086 |
| cg12585943 |
| cg01072821 |
| cg16832407 |
| cg02510853 |
| cg13226591 |
| cg14078518 |
| cg08088390 |
| cg24642523 |
| cg12874092 |
| cg17982102 |
| cg02037013 |
| cg19324313 |
| cg23234999 |
| cg13125510 |
| cg04349727 |
| cg00298951 |
| cg22815110 |
| cg19779211 |
| cg26389232 |
| cg21475402 |
| cg24346637 |
| cg02643667 |
| cg18129786 |
| cg06517798 |
| cg14120879 |
| cg15379633 |
| cg09585781 |
| cg21846488 |
| cg25107791 |
| cg19884600 |
| cg19279346 |
| cg02431964 |
| cg11801374 |
| cg18462653 |
| cg03221619 |
| cg21045388 |
| cg24505375 |
| cg02286642 |
| cg25058957 |
| cg22543128 |
| cg01076838 |
| cg03789934 |
| cg23370883 |
| cg15043057 |
| cg26872564 |
| cg06268694 |
| cg23765993 |
| cg27341860 |
| cg17978274 |
| cg27622610 |
| cg04481779 |
| cg13994177 |
| cg16673198 |
| cg04590978 |
| cg03597525 |
| cg17204100 |
| cg02723372 |
| cg16242770 |
| cg11828089 |
| cg11682508 |
| cg04164824 |
| cg03954858 |
| cg14544583 |
| cg11884243 |
| cg06222800 |
| cg10920765 |
| cg22456522 |
| cg27496506 |
| cg18484189 |
| cg07935568 |
| cg10198932 |
| cg07706362 |
| cg25177139 |
| cg01668383 |
| cg13158571 |
| cg25328975 |
| cg24489034 |
| cg24898863 |
| cg23205633 |
| cg10758292 |
| cg01204985 |
| cg07792737 |
| cg05174079 |
| cg18729973 |
| cg20576002 |
| cg13143729 |
| cg03417466 |
| cg15711744 |
| cg06066303 |
| cg23018448 |
| cg10766289 |
| cg11591325 |
| cg02701137 |
| cg06812844 |
| cg04443324 |
| cg22262964 |
| cg09546307 |
| cg10539808 |
| cg05828624 |
| cg08109815 |
| cg01558777 |
| cg03044435 |
| cg17063201 |
| cg15589427 |
| cg07109801 |
| cg02978737 |
| cg19685066 |
| cg25509184 |
| cg12791465 |
| cg00269932 |
| cg05248470 |
| cg06806711 |
| cg25650811 |
| cg21307628 |
| cg24357161 |
| cg11058932 |
| cg19138960 |
| cg07042144 |
| cg22954265 |
| cg21634602 |
| cg22643217 |
| cg11151665 |
| cg18201198 |
| cg23610820 |
| cg13530039 |
| cg21044104 |
| cg17628717 |
| cg14127336 |
| cg06521852 |
| cg25391023 |
| cg06816106 |
| cg25112191 |
| cg06118312 |
| cg01442426 |
| cg16786703 |
| cg11801011 |
| cg24498554 |
| cg00504595 |
| cg03167883 |
| cg17501569 |
| cg01870826 |
| cg17819635 |
| cg15417244 |
| cg25464840 |
| cg05799317 |
| cg00466436 |
| cg13644052 |
| cg23767977 |
| cg13482233 |
| cg24926042 |
| cg26879282 |
| cg16825000 |
| cg20182358 |
| cg27108154 |
| cg09558502 |
| cg23696712 |
| cg08459368 |
| cg25007250 |
| cg21919219 |
| cg07597976 |
| cg11846236 |
| cg24607535 |
| cg09923671 |
| cg15207953 |
| cg27389185 |
| cg27168844 |
| cg23244913 |
| cg23278885 |
| cg00319692 |
| cg25607161 |
| cg07373172 |
| cg20998885 |
| cg13899108 |
| cg24812103 |
| cg21457804 |
| cg22881914 |
| cg00745543 |
| cg03311899 |
| cg01669948 |
| cg16899306 |
| cg00397545 |
| cg13097816 |
| cg13577076 |
| cg20676303 |
| cg13379763 |
| cg07830847 |
| cg15494458 |
| cg16191009 |
