# Supplementary material for: 14-CpG-Based Signature Improves the Prognosis Prediction of Hepatocellular Carcinoma Patients
Source: Biomed Res Int. 2020 Jan 4;2020:9762067. doi: 10.1155/2020/9762067 (PMC6970499; doi:10.1155/2020/9762067)
Supplement: Supplementary Materials — Supplementary material 1: top 1000 differential CpGs between primary HCC tumors and their corresponding nontumor counterparts of GSE37988. Supplementary material 2: top 1000 differential CpGs between primary HCC tumors and their corresponding nontumor counterparts of GSE57958. Supplementary material 3: top 1000 differential CpGs between primary HCC tumors and their corresponding nontumor counterparts of GSE73003. Supplementary material 4: overlap of each top 1000 differential CpGs from GSE73003, GSE37988, and GSE57958 (426markers). Supplementary material 5: validated differential CpGs of GSE73003, GSE37988, and GSE57958 in HCC patients of TCGA (288 markers). [file 9762067.f1.zip › 9762067.f1/supplementary material 3.docx]

**Top 1000 differential CpGs between primary HCC tumors and their corresponding non-tumor counterparts of GSE73003**

| cg24816455 |
| --- |
| cg21790626 |
| cg06353345 |
| cg03975694 |
| cg25944100 |
| cg09120035 |
| cg25764191 |
| cg18766755 |
| cg04574507 |
| cg21643045 |
| cg25093045 |
| cg04600618 |
| cg00891278 |
| cg04349727 |
| cg20312687 |
| cg15606663 |
| cg12547930 |
| cg16744741 |
| cg07022477 |
| cg03872376 |
| cg04786857 |
| cg03616357 |
| cg06437004 |
| cg15149938 |
| cg05023540 |
| cg02755525 |
| cg08668790 |
| cg24432073 |
| cg12348970 |
| cg01309152 |
| cg17031773 |
| cg22643217 |
| cg26550234 |
| cg03504701 |
| cg13897627 |
| cg12078929 |
| cg27513764 |
| cg27043873 |
| cg05488632 |
| cg07014174 |
| cg07459489 |
| cg03960217 |
| cg25607161 |
| cg24304714 |
| cg14062083 |
| cg06906435 |
| cg15403517 |
| cg05684891 |
| cg23391785 |
| cg06244417 |
| cg16812893 |
| cg07548313 |
| cg05440289 |
| cg27071517 |
| cg07947016 |
| cg26066361 |
| cg25856811 |
| cg21431536 |
| cg07950803 |
| cg09748960 |
| cg00152644 |
| cg12891678 |
| cg17483510 |
| cg15016628 |
| cg15329483 |
| cg13139843 |
| cg06470471 |
| cg10521852 |
| cg19856444 |
| cg14826683 |
| cg08088390 |
| cg24169915 |
| cg02994956 |
| cg09478478 |
| cg26059632 |
| cg23776892 |
| cg06256735 |
| cg03243946 |
| cg00895324 |
| cg16431978 |
| cg05252264 |
| cg04505023 |
| cg07703401 |
| cg07374637 |
| cg06392096 |
| cg10707565 |
| cg23018448 |
| cg02764897 |
| cg25119415 |
| cg08555657 |
| cg04995095 |
| cg18780284 |
| cg17271365 |
| cg01546430 |
| cg15552238 |
| cg25384595 |
| cg00474004 |
| cg23338195 |
| cg00918005 |
| cg11554507 |
| cg14141399 |
| cg00466436 |
| cg01808508 |
| cg25902889 |
| cg23163573 |
| cg10370591 |
| cg19884600 |
| cg12014417 |
| cg20972553 |
| cg09702010 |
| cg15494458 |
| cg15747595 |
| cg15761405 |
| cg19226099 |
| cg01870826 |
| cg12133004 |
| cg01193293 |
| cg11119884 |
| cg06952310 |
| cg12493906 |
| cg12970081 |
| cg01772980 |
| cg19623751 |
| cg01668126 |
| cg09786257 |
| cg24824840 |
| cg02946850 |
| cg24765446 |
| cg03014957 |
| cg03914397 |
| cg04103514 |
| cg02311163 |
| cg11061975 |
| cg11015241 |
| cg15746620 |
| cg10503138 |
| cg22424444 |
| cg02148642 |
| cg01144251 |
| cg04721098 |
| cg02833180 |
| cg00503840 |
| cg22815110 |
| cg17405586 |
| cg22951794 |
| cg25072962 |
| cg04962134 |
| cg02936263 |
| cg00705255 |
| cg24352499 |
| cg19787037 |
| cg13792279 |
| cg08970694 |
| cg22767466 |
| cg08321346 |
| cg05248470 |
| cg17560332 |
| cg23029519 |
| cg00138126 |
| cg25608041 |
| cg07259382 |
| cg20305726 |
| cg27160701 |
| cg27009703 |
| cg08590939 |
| cg15610233 |
| cg07378350 |
| cg11520395 |
| cg18967533 |
| cg03602500 |
| cg11698653 |
| cg00958560 |
| cg06263495 |
| cg08786003 |
| cg27344326 |
| cg26861460 |
| cg24736099 |
| cg24870391 |
| cg25082710 |
| cg05126264 |
| cg20070090 |
| cg17357062 |
| cg03020951 |
| cg27389185 |
| cg11377136 |
| cg18873386 |
| cg03213216 |
| cg12339029 |
| cg22984277 |
| cg17173423 |
| cg04983977 |
| cg25612480 |
| cg04484789 |
| cg08458170 |
| cg22477971 |
| cg19784470 |
| cg07747336 |
| cg09076077 |
| cg23937047 |
| cg08260959 |
| cg02677802 |
| cg00891541 |
| cg00967316 |
| cg21754343 |
| cg19554294 |
| cg00463848 |
| cg21529807 |
| cg12680609 |
| cg07706362 |
| cg20649991 |
| cg23032612 |
| cg21930712 |
| cg08886154 |
| cg25098401 |
| cg00948524 |
| cg07711097 |
| cg26029902 |
| cg23001457 |
| cg05832051 |
| cg15821095 |
| cg22113807 |
| cg03941108 |
| cg15952487 |
| cg12460359 |
| cg08831522 |
| cg21023114 |
| cg24387380 |
| cg15014458 |
| cg26738880 |
| cg02909790 |
| cg03417466 |
| cg12150401 |
| cg03544379 |
| cg02284188 |
| cg16742703 |
| cg10807560 |
| cg04958389 |
| cg14988503 |
| cg22190114 |
| cg11710560 |
| cg26113512 |
| cg22039287 |
| cg06117855 |
| cg25033144 |
| cg23307338 |
| cg27429194 |
| cg12108912 |
| cg08878744 |
| cg11843304 |
| cg09828634 |
| cg01543654 |
| cg19421752 |
| cg01731341 |
| cg00116838 |
| cg09099744 |
| cg18841952 |
| cg02721374 |
| cg16122592 |
| cg14738823 |
| cg24812103 |
| cg10533434 |
| cg03898365 |
| cg06434470 |
| cg26090660 |
| cg08099701 |
| cg17301902 |
| cg18457737 |
| cg04545516 |
| cg04920951 |
| cg13158571 |
| cg23413307 |
| cg26164184 |
| cg14310034 |
| cg19306866 |
| cg03116740 |
| cg13853761 |
| cg05779068 |
| cg09558502 |
| cg24027342 |
| cg13697387 |
| cg25890048 |
| cg07384961 |
| cg09532664 |
| cg09453312 |
| cg19797376 |
| cg16673198 |
| cg08209133 |
| cg24468890 |
| cg01906717 |
| cg13462129 |
| cg06621358 |
| cg07745725 |
| cg23248452 |
| cg03468463 |
| cg25462303 |
| cg00401678 |
| cg24489034 |
| cg25450806 |
| cg24272907 |
| cg17928268 |
| cg19863740 |
| cg21065959 |
| cg27351998 |
| cg08539093 |
| cg20542190 |
| cg04645843 |
| cg13801416 |
| cg20312228 |
| cg11059341 |
| cg26104469 |
| cg10377274 |
| cg26614346 |
| cg19418515 |
| cg02493771 |
| cg11750883 |
| cg19404979 |
| cg21434954 |
| cg14153740 |
| cg14696820 |
| cg02868123 |
| cg13300756 |
| cg22920417 |
| cg00564163 |
| cg19996355 |
| cg14940420 |
| cg04439215 |
| cg06222800 |
| cg08684473 |
| cg15092802 |
| cg05810550 |
| cg06639544 |
| cg12315311 |
| cg14576824 |
| cg01532771 |
| cg00495491 |
| cg06101324 |
| cg15375239 |
| cg21243096 |
| cg13143729 |
| cg19356189 |
| cg18822544 |
| cg03029616 |
| cg14456683 |
| cg25259754 |
| cg02423618 |
| cg02332073 |
| cg04138756 |
| cg03789934 |
| cg20998885 |
| cg05073035 |
| cg01295203 |
| cg13928961 |
| cg05364569 |
| cg14417329 |
| cg10977115 |
| cg04164824 |
| cg15044041 |
| cg09555879 |
| cg09492887 |
| cg21312148 |
| cg18338293 |
| cg06123346 |
| cg23581186 |
| cg00750606 |
| cg15811427 |
| cg10371914 |
| cg25322008 |
| cg09422355 |
| cg00548268 |
| cg15792367 |
| cg15415545 |
| cg22646937 |
| cg15669228 |
| cg25388528 |
| cg17886204 |
| cg03329572 |
| cg09546307 |
| cg26829529 |
| cg10766289 |
| cg19553463 |
| cg17687962 |
| cg10575735 |
| cg15531099 |
| cg03958979 |
| cg21607649 |
| cg04711324 |
| cg05782445 |
| cg19324313 |
| cg13164309 |
| cg11846968 |
| cg23187653 |
| cg21825364 |
| cg15602735 |
| cg08097882 |
| cg25391023 |
| cg23407366 |
| cg24063382 |
| cg11959435 |
| cg13758677 |
| cg26465666 |
| cg07038400 |
| cg10677144 |
| cg12237269 |
| cg24816298 |
| cg06605933 |
| cg17200465 |
| cg02124291 |
| cg03109316 |
| cg13407883 |
| cg02510853 |
| cg25802093 |
| cg15670863 |
| cg21284731 |
| cg25839766 |
| cg25340403 |
| cg15657668 |
| cg26565975 |
| cg04809136 |
| cg16242770 |
| cg06811800 |
| cg26813458 |
| cg15379633 |
| cg16466334 |
| cg18956481 |
| cg11435943 |
| cg15538820 |
| cg01598642 |
| cg08763351 |
| cg24992780 |
| cg19982860 |
| cg20145360 |
| cg18462653 |
| cg21414251 |
| cg14757492 |
| cg27341860 |
| cg26718420 |
| cg23984130 |
| cg26087862 |
| cg09786221 |
| cg03561565 |
| cg01796228 |
| cg20769842 |
| cg07373172 |
| cg21902327 |
| cg06226384 |
| cg02164046 |
| cg15842276 |
| cg25697314 |
| cg13984181 |
| cg14544583 |
| cg01999333 |
| cg02655204 |
| cg12281657 |
| cg08981777 |
| cg22740835 |
| cg24884084 |
| cg00949442 |
| cg01055695 |
| cg13798289 |
| cg17738194 |
| cg00187686 |
| cg14911395 |
| cg10335112 |
| cg06784466 |
| cg18982568 |
| cg16689634 |
| cg24484296 |
| cg07841014 |
| cg00427635 |
| cg12513379 |
| cg15209169 |
| cg24377133 |
| cg04034767 |
| cg07136161 |
| cg18484189 |
| cg01871995 |
| cg01868128 |
| cg19388557 |
| cg15737319 |
| cg09847584 |
| cg13899108 |
| cg18343292 |
| cg14162076 |
| cg08214957 |
| cg17327492 |
| cg10094277 |
| cg03673470 |
| cg25864727 |
| cg21045388 |
| cg26264314 |
| cg18849169 |
| cg21578906 |
| cg14353201 |
| cg10127415 |
| cg15741706 |
| cg23743472 |
| cg10691387 |
| cg21307628 |
| cg05828624 |
| cg19464944 |
| cg01344171 |
| cg12188860 |
| cg19297232 |
| cg03515901 |
| cg09845785 |
| cg01215061 |
| cg19216731 |
| cg14869028 |
| cg10412312 |
| cg22855405 |
| cg17356112 |
| cg00504595 |
| cg11673092 |
| cg20018806 |
| cg03826976 |
| cg07717632 |
| cg11068096 |
| cg10978355 |
| cg09617773 |
| cg09841009 |
| cg26312920 |
| cg06319346 |
| cg26848126 |
| cg09260089 |
| cg07409200 |
| cg17205788 |
| cg06318853 |
| cg14444710 |
| cg26776077 |
| cg12379145 |
| cg24300924 |
| cg02440177 |
| cg20833786 |
| cg24107142 |
| cg00406188 |
| cg14704941 |
| cg22836229 |
| cg24898863 |
| cg22809047 |
| cg03977657 |
| cg03705396 |
| cg08713365 |
| cg07297178 |
| cg01074640 |
| cg12038710 |
| cg27420123 |
| cg01381846 |
| cg08441806 |
| cg23642747 |
| cg06799664 |
| cg25107903 |
| cg23855121 |
| cg04111761 |
| cg04901273 |
| cg18952647 |
| cg20437604 |
| cg14613546 |
| cg01962826 |
| cg21457147 |
| cg11052143 |
| cg07922606 |
| cg27553955 |
| cg10920765 |
| cg11935147 |
| cg21349901 |
| cg17423978 |
| cg25214366 |
| cg20676475 |
| cg10556064 |
| cg19279346 |
| cg18129786 |
| cg23110514 |
| cg03741352 |
| cg17819635 |
| cg06291867 |
| cg17474651 |
| cg03732545 |
| cg26538442 |
| cg19593572 |
| cg16791686 |
| cg02620013 |
| cg05659947 |
| cg16678925 |
| cg14826456 |
| cg09868882 |
| cg27299588 |
| cg10269439 |
| cg11884243 |
| cg08268099 |
| cg11070419 |
| cg17657618 |
| cg04448487 |
| cg21512370 |
| cg17173856 |
| cg13745346 |
| cg16377872 |
| cg07654934 |
| cg22861316 |
| cg19686152 |
| cg01161611 |
| cg19384697 |
| cg23623692 |
| cg18815943 |
| cg10853416 |
| cg07545232 |
| cg11173246 |
| cg01713938 |
| cg13003163 |
| cg06084117 |
| cg23350580 |
| cg10198932 |
| cg17982102 |
| cg20822579 |
| cg03070194 |
| cg12532667 |
| cg03292149 |
| cg11812202 |
| cg27000831 |
| cg08704606 |
| cg07403350 |
| cg18149919 |
| cg19140639 |
| cg18555440 |
| cg17725968 |
| cg09174741 |
| cg26895595 |
| cg03469158 |
| cg10981541 |
| cg19803984 |
| cg24546942 |
| cg27416067 |
| cg00601486 |
| cg18674980 |
| cg01076838 |
| cg15873301 |
| cg00280894 |
| cg16953612 |
| cg02593766 |
| cg15589427 |
| cg04070847 |
| cg16303562 |
| cg15408454 |
| cg03902905 |
| cg25268283 |
| cg00579393 |
| cg18509435 |
| cg02919422 |
| cg24477636 |
| cg10246520 |
| cg14533138 |
| cg20909686 |
| cg03716937 |
| cg04086012 |
| cg24217877 |
| cg18236477 |
| cg27456885 |
| cg01568736 |
| cg16872071 |
| cg01796223 |
| cg22268164 |
| cg23595927 |
| cg16899306 |
| cg18885299 |
| cg10129493 |
| cg20311730 |
| cg19283196 |
| cg02813121 |
| cg21282997 |
| cg17118262 |
| cg20125091 |
| cg25133685 |
| cg23818978 |
| cg20950277 |
| cg21044104 |
| cg18279742 |
| cg02202484 |
| cg14021698 |
| cg02037013 |
| cg16970232 |
| cg12200412 |
| cg00627233 |
| cg24861272 |
| cg20349377 |
| cg06194186 |
| cg12365667 |
| cg13693652 |
| cg19345602 |
| cg19782598 |
| cg16617137 |
| cg14284171 |
| cg22375192 |
| cg23941599 |
| cg17281600 |
| cg18534730 |
| cg22631938 |
| cg16786703 |
| cg21846488 |
| cg07752420 |
| cg10379687 |
| cg06806080 |
| cg08460435 |
| cg08489623 |
| cg23244913 |
| cg03312792 |
| cg16514843 |
| cg16158220 |
| cg11328541 |
| cg03780486 |
| cg20080624 |
| cg01549015 |
| cg03818682 |
| cg13410437 |
| cg05535113 |
| cg06748315 |
| cg10978346 |
| cg24607535 |
| cg17470143 |
| cg18680834 |
| cg06208111 |
| cg27460755 |
| cg20119871 |
| cg24401441 |
| cg13707560 |
| cg23410113 |
| cg23865698 |
| cg13246269 |
| cg02280309 |
| cg19290962 |
| cg24423088 |
| cg15869642 |
| cg25957124 |
| cg01204985 |
| cg12682367 |
| cg12728629 |
| cg27626299 |
| cg00476577 |
| cg04052038 |
| cg08583049 |
| cg11801011 |
| cg07864297 |
| cg24624841 |
| cg07830847 |
| cg14236389 |
| cg20073553 |
| cg16998872 |
| cg15868302 |
| cg01612158 |
| cg25650811 |
| cg06293195 |
| cg20256783 |
| cg04774694 |
| cg18572014 |
| cg02659086 |
| cg17063201 |
| cg10523494 |
| cg01683883 |
| cg12762799 |
| cg24110050 |
| cg16131766 |
| cg23092823 |
| cg17036737 |
| cg08935003 |
| cg11724759 |
| cg16542081 |
| cg08458487 |
| cg16218254 |
| cg06993413 |
| cg10321723 |
| cg13019092 |
| cg12718562 |
| cg09038676 |
| cg22983092 |
| cg08124722 |
| cg01909378 |
| cg10725344 |
| cg16636571 |
| cg21003606 |
| cg20977864 |
| cg00546897 |
| cg06268694 |
| cg04000821 |
| cg02601403 |
| cg17827767 |
| cg04345908 |
| cg21707816 |
| cg02784874 |
| cg04431776 |
| cg08872742 |
| cg15983005 |
| cg09643544 |
| cg12582965 |
| cg11846236 |
| cg27214365 |
| cg11653709 |
| cg21462299 |
| cg13226591 |
| cg18671950 |
| cg19297688 |
| cg01714513 |
| cg26063872 |
| cg22197708 |
| cg26207503 |
| cg12758687 |
| cg10938286 |
| cg23158811 |
| cg05221167 |
| cg23659398 |
| cg05949173 |
| cg16386158 |
| cg15988792 |
| cg15175266 |
| cg07236769 |
| cg08840010 |
| cg16192029 |
| cg05521696 |
| cg23765993 |
| cg12582008 |
| cg05767404 |
| cg07054641 |
| cg15602809 |
| cg02504280 |
| cg16612562 |
| cg03167883 |
| cg16797831 |
| cg04590978 |
| cg25229305 |
| cg13694749 |
| cg06806711 |
| cg14119236 |
| cg04663487 |
| cg20485165 |
| cg02255732 |
| cg15787039 |
| cg24642523 |
| cg26499286 |
| cg04336379 |
| cg24875415 |
| cg21604803 |
| cg09768051 |
| cg24357161 |
| cg03742272 |
| cg20556988 |
| cg24355048 |
| cg14030359 |
| cg09952204 |
| cg11471401 |
| cg03876618 |
| cg16413535 |
| cg21474838 |
| cg08263647 |
| cg14535518 |
| cg23696618 |
| cg18342279 |
| cg23458892 |
| cg21991396 |
| cg09649610 |
| cg22478614 |
| cg17740305 |
| cg02992767 |
| cg13279585 |
| cg05112299 |
| cg26301777 |
| cg25509184 |
| cg24940601 |
| cg01109219 |
| cg01248426 |
| cg07973461 |
| cg26333641 |
| cg20308679 |
| cg16483466 |
| cg03802191 |
| cg18020749 |
| cg24949488 |
| cg16029760 |
| cg14659547 |
| cg08801754 |
| cg03283694 |
| cg10746737 |
| cg16670497 |
| cg06233985 |
| cg11337780 |
| cg03127543 |
| cg18499731 |
| cg23410627 |
| cg27090216 |
| cg12555907 |
| cg10576828 |
| cg02442161 |
| cg18536148 |
| cg22995176 |
| cg13547644 |
| cg18806980 |
| cg16016036 |
| cg25372195 |
| cg13633026 |
| cg07233761 |
| cg00746981 |
| cg20114394 |
| cg06230736 |
| cg15627025 |
| cg17092637 |
| cg21256656 |
| cg11344614 |
| cg10751811 |
| cg06613840 |
| cg14415300 |
| cg06154597 |
| cg26112639 |
| cg20092728 |
| cg15746445 |
| cg03521113 |
| cg25545210 |
| cg27168844 |
| cg06073471 |
| cg14312526 |
| cg10078829 |
| cg16362133 |
| cg10399228 |
| cg15520279 |
| cg20520725 |
| cg21671476 |
| cg22496652 |
| cg11500797 |
| cg01375871 |
| cg04446579 |
| cg16372520 |
| cg19955257 |
| cg05636175 |
| cg15228639 |
| cg07484450 |
| cg24653181 |
| cg24926780 |
| cg00347904 |
| cg11270633 |
| cg04731384 |
| cg11880010 |
| cg27016494 |
| cg24851490 |
| cg18901980 |
| cg25737664 |
| cg15316334 |
| cg12998491 |
| cg05799317 |
| cg03017475 |
| cg16832407 |
| cg13878010 |
| cg22495124 |
| cg20609368 |
| cg19560971 |
| cg03473518 |
| cg11102782 |
| cg08420900 |
| cg14916079 |
| cg10451565 |
| cg08284151 |
| cg16411152 |
| cg15928132 |
| cg24784109 |
| cg07703337 |
| cg21023001 |
| cg14143055 |
| cg05393484 |
| cg13471990 |
| cg02812142 |
| cg20557104 |
| cg09794131 |
| cg14399656 |
| cg23679141 |
| cg16112157 |
| cg12588301 |
| cg21870884 |
| cg16324018 |
| cg11122968 |
| cg05039054 |
| cg26561773 |
| cg21039631 |
| cg01469547 |
| cg01637734 |
| cg25692621 |
| cg10671066 |
| cg00229387 |
| cg20786074 |
| cg00983899 |
| cg19258973 |
| cg20022541 |
| cg18986165 |
| cg14011639 |
| cg05554936 |
| cg17866455 |
| cg20047055 |
| cg10895543 |
| cg25781202 |
| cg26609631 |
| cg26090652 |
| cg22131172 |
| cg02830467 |
| cg08996986 |
| cg20182358 |
| cg02475653 |
| cg15761233 |
