# Supplementary material for: 14-CpG-Based Signature Improves the Prognosis Prediction of Hepatocellular Carcinoma Patients
Source: Biomed Res Int. 2020 Jan 4;2020:9762067. doi: 10.1155/2020/9762067 (PMC6970499; doi:10.1155/2020/9762067)
Supplement: Supplementary Materials — Supplementary material 1: top 1000 differential CpGs between primary HCC tumors and their corresponding nontumor counterparts of GSE37988. Supplementary material 2: top 1000 differential CpGs between primary HCC tumors and their corresponding nontumor counterparts of GSE57958. Supplementary material 3: top 1000 differential CpGs between primary HCC tumors and their corresponding nontumor counterparts of GSE73003. Supplementary material 4: overlap of each top 1000 differential CpGs from GSE73003, GSE37988, and GSE57958 (426markers). Supplementary material 5: validated differential CpGs of GSE73003, GSE37988, and GSE57958 in HCC patients of TCGA (288 markers). [file 9762067.f1.zip › 9762067.f1/supplementary material 4.docx]

**Overlap of each top 1000 differential CpGs from GSE73003, GSE37988 and GSE57958 (426markers)**

cg01868128

cg27071517

cg06392096

cg08268099

cg14826683

cg04431776

cg13928961

cg09076077

cg16431978

cg24352499

cg02148642

cg22190114

cg16953612

cg05252264

cg11801011

cg08539093

cg04962134

cg12339029

cg01772980

cg15014458

cg02721374

cg11698653

cg07374637

cg12728629

cg08441806

cg17357062

cg05659947

cg21991396

cg15952487

cg19216731

cg02423618

cg05488632

cg21578906

cg27389185

cg02601403

cg09099744

cg20256783

cg25093045

cg13407883

cg14153740

cg18766755

cg20073553

cg23642747

cg02332073

cg07950803

cg13745346

cg07747336

cg08878744

cg15092802

cg01469547

cg07745725

cg07545232

cg07706362

cg23776892

cg10920765

cg17687962

cg20047055

cg19279346

cg20070090

cg10807560

cg17738194

cg12348970

cg11750883

cg02868123

cg24432073

cg23001457

cg20182358

cg02311163

cg08872742

cg25119415

cg15787039

cg15821095

cg02909790

cg18780284

cg08124722

cg00895324

cg00601486

cg17327492

cg13897627

cg25372195

cg15627025

cg10707565

cg27043873

cg04138756

cg08668790

cg27344326

cg18534730

cg15746620

cg22951794

cg22268164

cg23018448

cg15329483

cg19863740

cg19356189

cg00463848

cg06244417

cg05684891

cg00152644

cg25033144

cg03602500

cg13792279

cg20119871

cg03914397

cg04034767

cg25340403

cg10129493

cg23595927

cg14704941

cg02784874

cg12680609

cg09847584

cg17405586

cg10691387

cg04731384

cg04645843

cg23413307

cg03504701

cg03789934

cg01962826

cg05767404

cg17474651

cg11653709

cg21790626

cg21643045

cg04349727

cg03872376

cg21434954

cg12108912

cg08555657

cg20305726

cg08684473

cg12150401

cg11959435

cg26776077

cg18873386

cg03818682

cg15669228

cg09555879

cg07014174

cg25545210

cg24870391

cg21023114

cg01144251

cg16192029

cg12200412

cg03975694

cg14415300

cg24607535

cg19226099

cg05799317

cg12970081

cg17886204

cg18462653

cg18849169

cg01076838

cg04995095

cg25098401

cg15408454

cg03312792

cg07373172

cg24898863

cg12493906

cg16673198

cg02677802

cg22767466

cg05779068

cg24423088

cg16016036

cg25462303

cg27016494

cg18841952

cg15494458

cg24824840

cg17827767

cg24107142

cg10370591

cg02593766

cg19464944

cg08981777

cg03109316

cg03741352

cg17356112

cg01808508

cg26063872

cg07297178

cg01375871

cg20542190

cg26829529

cg06101324

cg19996355

cg14544583

cg16617137

cg16242770

cg17982102

cg27513764

cg04711324

cg11015241

cg24736099

cg19345602

cg14659547

cg01055695

cg00138126

cg25214366

cg11884243

cg07752420

cg16303562

cg01074640

cg20311730

cg12188860

cg26059632

cg17928268

cg06639544

cg12547930

cg14444710

cg11846968

cg03213216

cg25839766

cg22477971

cg21902327

cg11843304

cg21045388

cg16514843

cg00891278

cg17173423

cg08886154

cg23350580

cg21825364

cg01731341

cg14988503

cg19306866

cg07409200

cg04600618

cg16744741

cg13226591

cg10895543

cg03544379

cg14911395

cg07973461

cg14284171

cg11935147

cg19290962

cg14533138

cg15983005

cg24169915

cg25384595

cg07841014

cg24355048

cg22646937

cg24765446

cg08458170

cg12718562

cg01309152

cg25612480

cg22478614

cg07022477

cg06226384

cg15602735

cg11435943

cg23338195

cg03716937

cg24642523

cg13899108

cg15552238

cg11710560

cg06291867

cg01193293

cg06256735

cg08260959

cg20998885

cg24861272

cg10766289

cg01637734

cg04574507

cg00504595

cg20649991

cg14310034

cg04721098

cg11377136

cg06263495

cg17560332

cg22643217

cg27214365

cg00918005

cg22815110

cg03014957

cg19421752

cg21307628

cg11554507

cg09617773

cg12891678

cg07459489

cg16998872

cg20312687

cg06952310

cg25107903

cg26090660

cg15538820

cg14141399

cg12014417

cg10575735

cg05832051

cg06437004

cg14062083

cg20485165

cg08970694

cg13694749

cg06806711

cg24304714

cg10335112

cg02442161

cg14162076

cg08088390

cg00466436

cg03283694

cg00546897

cg05126264

cg27553955

cg13158571

cg24063382

cg02037013

cg10576828

cg08097882

cg08786003

cg04000821

cg22861316

cg11061975

cg14940420

cg00949442

cg07654934

cg04439215

cg26813458

cg08460435

cg12682367

cg04164824

cg13164309

cg17657618

cg26164184

cg16122592

cg09120035

cg24816455

cg23743472

cg24812103

cg20018806

cg05023540

cg25856811

cg06353345

cg18555440

cg11068096

cg07548313

cg13758677

cg07947016

cg27420123

cg10127415

cg01204985

cg19856444

cg01668126

cg26738880

cg00750606

cg19623751

cg10198932

cg22424444

cg19982860

cg04786857

cg07711097

cg14236389

cg25607161

cg05440289

cg23865698

cg21930712

cg25072962

cg06906435

cg01215061

cg00705255

cg15747595

cg23391785

cg26312920

cg05521696

cg25082710

cg15842276

cg16899306

cg25764191

cg04086012

cg13471990

cg25259754

cg14757492

cg06319346

cg18343292

cg03941108

cg09260089

cg04505023

cg23984130

cg10853416

cg11500797

cg00280894

cg25509184

cg10503138

cg18129786

cg16678925

cg18484189

cg25391023

cg01598642

cg17205788

cg15531099

cg19787037

cg02812142

cg15670863

cg23163573

cg18967533

cg03742272

cg09841009

cg06811800

cg04484789

cg15741706

cg15606663

cg05828624

cg08583049

cg16812893

cg13300756

cg12315311

cg09558502

cg02284188

cg19140639

cg18536148

cg04901273

cg04345908
