# Supplementary material for: 14-CpG-Based Signature Improves the Prognosis Prediction of Hepatocellular Carcinoma Patients
Source: Biomed Res Int. 2020 Jan 4;2020:9762067. doi: 10.1155/2020/9762067 (PMC6970499; doi:10.1155/2020/9762067)
Supplement: Supplementary Materials — Supplementary material 1: top 1000 differential CpGs between primary HCC tumors and their corresponding nontumor counterparts of GSE37988. Supplementary material 2: top 1000 differential CpGs between primary HCC tumors and their corresponding nontumor counterparts of GSE57958. Supplementary material 3: top 1000 differential CpGs between primary HCC tumors and their corresponding nontumor counterparts of GSE73003. Supplementary material 4: overlap of each top 1000 differential CpGs from GSE73003, GSE37988, and GSE57958 (426markers). Supplementary material 5: validated differential CpGs of GSE73003, GSE37988, and GSE57958 in HCC patients of TCGA (288 markers). [file 9762067.f1.zip › 9762067.f1/supplementary material 5.docx]

**Validated differential CpGs of GSE73003, GSE37988 and GSE57958 in HCC patients of TCGA (288 markers)**

| cg27071517 |
| --- |
| cg08268099 |
| cg14826683 |
| cg13928961 |
| cg09076077 |
| cg16431978 |
| cg22190114 |
| cg16953612 |
| cg05252264 |
| cg11801011 |
| cg04962134 |
| cg01772980 |
| cg15014458 |
| cg02721374 |
| cg07374637 |
| cg08441806 |
| cg17357062 |
| cg05659947 |
| cg21991396 |
| cg15952487 |
| cg19216731 |
| cg02423618 |
| cg05488632 |
| cg21578906 |
| cg20256783 |
| cg25093045 |
| cg13407883 |
| cg14153740 |
| cg18766755 |
| cg20073553 |
| cg23642747 |
| cg07950803 |
| cg13745346 |
| cg01469547 |
| cg07745725 |
| cg07706362 |
| cg17687962 |
| cg19279346 |
| cg20070090 |
| cg10807560 |
| cg17738194 |
| cg12348970 |
| cg11750883 |
| cg24432073 |
| cg23001457 |
| cg20182358 |
| cg02311163 |
| cg08872742 |
| cg25119415 |
| cg15787039 |
| cg15821095 |
| cg02909790 |
| cg00895324 |
| cg00601486 |
| cg17327492 |
| cg13897627 |
| cg25372195 |
| cg10707565 |
| cg04138756 |
| cg08668790 |
| cg15746620 |
| cg22268164 |
| cg15329483 |
| cg19863740 |
| cg00463848 |
| cg06244417 |
| cg05684891 |
| cg00152644 |
| cg25033144 |
| cg03602500 |
| cg13792279 |
| cg20119871 |
| cg03914397 |
| cg04034767 |
| cg25340403 |
| cg10129493 |
| cg14704941 |
| cg02784874 |
| cg09847584 |
| cg17405586 |
| cg10691387 |
| cg04731384 |
| cg23413307 |
| cg05767404 |
| cg21790626 |
| cg21643045 |
| cg04349727 |
| cg03872376 |
| cg12108912 |
| cg08555657 |
| cg20305726 |
| cg08684473 |
| cg12150401 |
| cg18873386 |
| cg03818682 |
| cg15669228 |
| cg09555879 |
| cg07014174 |
| cg24870391 |
| cg16192029 |
| cg12200412 |
| cg24607535 |
| cg19226099 |
| cg05799317 |
| cg12970081 |
| cg18462653 |
| cg18849169 |
| cg01076838 |
| cg25098401 |
| cg24898863 |
| cg12493906 |
| cg16673198 |
| cg24423088 |
| cg16016036 |
| cg25462303 |
| cg27016494 |
| cg18841952 |
| cg24824840 |
| cg17827767 |
| cg10370591 |
| cg02593766 |
| cg08981777 |
| cg03109316 |
| cg17356112 |
| cg07297178 |
| cg01375871 |
| cg20542190 |
| cg26829529 |
| cg06101324 |
| cg19996355 |
| cg14544583 |
| cg17982102 |
| cg04711324 |
| cg11015241 |
| cg19345602 |
| cg14659547 |
| cg01055695 |
| cg00138126 |
| cg11884243 |
| cg16303562 |
| cg20311730 |
| cg12188860 |
| cg26059632 |
| cg17928268 |
| cg12547930 |
| cg11846968 |
| cg03213216 |
| cg22477971 |
| cg21902327 |
| cg21045388 |
| cg16514843 |
| cg00891278 |
| cg17173423 |
| cg08886154 |
| cg23350580 |
| cg01731341 |
| cg14988503 |
| cg19306866 |
| cg07409200 |
| cg04600618 |
| cg16744741 |
| cg13226591 |
| cg03544379 |
| cg14911395 |
| cg11935147 |
| cg19290962 |
| cg15983005 |
| cg24169915 |
| cg25384595 |
| cg07841014 |
| cg24355048 |
| cg24765446 |
| cg08458170 |
| cg25612480 |
| cg06226384 |
| cg15602735 |
| cg11435943 |
| cg23338195 |
| cg03716937 |
| cg24642523 |
| cg13899108 |
| cg15552238 |
| cg06291867 |
| cg01193293 |
| cg08260959 |
| cg20998885 |
| cg24861272 |
| cg10766289 |
| cg01637734 |
| cg04574507 |
| cg00504595 |
| cg20649991 |
| cg14310034 |
| cg11377136 |
| cg06263495 |
| cg22643217 |
| cg27214365 |
| cg22815110 |
| cg19421752 |
| cg21307628 |
| cg11554507 |
| cg12891678 |
| cg07459489 |
| cg20312687 |
| cg06952310 |
| cg15538820 |
| cg14141399 |
| cg05832051 |
| cg06437004 |
| cg14062083 |
| cg20485165 |
| cg08970694 |
| cg13694749 |
| cg06806711 |
| cg24304714 |
| cg10335112 |
| cg08088390 |
| cg00546897 |
| cg27553955 |
| cg13158571 |
| cg02037013 |
| cg10576828 |
| cg08097882 |
| cg08786003 |
| cg22861316 |
| cg07654934 |
| cg04439215 |
| cg26813458 |
| cg08460435 |
| cg12682367 |
| cg17657618 |
| cg26164184 |
| cg16122592 |
| cg09120035 |
| cg24816455 |
| cg20018806 |
| cg25856811 |
| cg06353345 |
| cg07548313 |
| cg07947016 |
| cg10127415 |
| cg01204985 |
| cg19856444 |
| cg26738880 |
| cg10198932 |
| cg04786857 |
| cg07711097 |
| cg14236389 |
| cg25607161 |
| cg05440289 |
| cg23865698 |
| cg25072962 |
| cg06906435 |
| cg00705255 |
| cg15747595 |
| cg23391785 |
| cg26312920 |
| cg25082710 |
| cg15842276 |
| cg04086012 |
| cg13471990 |
| cg25259754 |
| cg18343292 |
| cg03941108 |
| cg09260089 |
| cg04505023 |
| cg11500797 |
| cg25509184 |
| cg10503138 |
| cg18129786 |
| cg16678925 |
| cg18484189 |
| cg25391023 |
| cg01598642 |
| cg17205788 |
| cg15670863 |
| cg23163573 |
| cg18967533 |
| cg03742272 |
| cg06811800 |
| cg04484789 |
| cg05828624 |
| cg08583049 |
| cg16812893 |
| cg13300756 |
| cg09558502 |
| cg18536148 |
| cg04345908 |
